# The frequency of medical reversals in a cross-sectional analysis of high-impact oncology journals, 2009–2018

**DOI:** 10.1186/s12885-021-08632-8

**Published:** 2021-08-03

**Authors:** Alyson Haslam, Jennifer Gill, Tyler Crain, Diana Herrera-Perez, Emerson Y. Chen, Talal Hilal, Myung S. Kim, Vinay Prasad

**Affiliations:** 1 Department of Epidemiology and Biostatistics, University of California, San Francisco, USA; 2 Providence Health & Services, Portland, OR USA; 3 Department of Analytics, Northwest Permanente, Portland, OR USA; 4 Oregon Health & Science University, Portland, OR USA; 5 University of Mississippi Medical Center, Jackson, MS USA

**Keywords:** Oncology, Medical reversal, Low-value care

## Abstract

**Background:**

Identifying ineffective practices that have been used in oncology is important in reducing wasted resources and harm. We sought to examine the prevalence of practices that are being used but have been shown in RCTs to be ineffective (medical reversals) in published oncology studies.

**Methods:**

We cross-sectionally analyzed studies published in three high-impact oncology medical journals (2009–2018). We abstracted data relating to the frequency and characterization of medical reversals.

**Results:**

Of the 64 oncology reversals, medications (44%) represented the most common intervention type (39% were targeted). Fourteen (22%) were funded by pharmaceutical/industry only and 56% were funded by an organization other than pharmaceutical/industry. The median number of years that the practice had been in use prior to the reversal study was 9 years (range 1–50 years).

**Conclusion:**

Here we show that oncology reversals most often involve the administration of medications, have been practiced for years, and are often identified through studies funded by non-industry organizations.

**Supplementary Information:**

The online version contains supplementary material available at 10.1186/s12885-021-08632-8.

## Introduction

Medical reversals, which are practices employed outside of clinical trials that are later found to be no better than a prior or lesser standard of care in a randomized controlled trial (RCT), are present in all medical disciplines [1]. Oncology is an important field to study the phenomenon of medical reversals, because it often includes the care of lethal and feared diseases, where patients and physicians may be willing to engage in treatments without support from prior RCTs, especially for individuals with advanced, metastatic or incurable cancers with few or no satisfactory treatment options [2].

If such practices are later validated, patients have benefit from early access to a promising intervention. If such practices are later refuted, costs and downsides include (a) opportunity costs, (b) side effects or off target effects of therapy, and (c) financial burden. Identifying ineffective practices that are being or have been used in oncology practice is especially important—not only to reduce wasted resources but to also reduce harm that often accompany ineffective practices.

We have previously characterized a group of almost 400 practices found in high impact factor medical journals, which are considered medical reversals [3], and of which approximately 6% were oncology practices. It is our purpose in this study to examine the prevalence of medical reversals in high impact factor oncology journals and to identify those specific practices that are being used but have been shown in RCTs to be ineffective.

## Methods

### Article inclusion

We reviewed all articles published in high impact factor oncology journals during a 10-year period – Lancet Oncology, Journal of Clinical Oncology (JCO), and Journal of the American Medical Association (JAMA) Oncology. The journals were chosen based on impact factor, specifically the 5-year Hirsch index for medical journals (https://jcr.clarivate.com/JCRJournalHomeAction.action?), for the year 2018 and those journals that published an average of at least 20 RCTs per year. From these articles, we selected all RCTs under the heading of “Articles” in Lancet Oncology, “Original Reports” in JCO, and “Original Investigation” for JAMA Oncology. Selected articles needed to report the results in the overall study population. We excluded articles that reported stratified or subgroup analysis only or that did not analyze the outcome in randomized groups. We also excluded articles that did not test a medical intervention or that were pharmacodynamics studies.

We used articles published between January 1, 2009 and December 31, 2018. Because JAMA Oncology’s inception was April 2015, we only looked at articles between then and December 31, 2018 for this journal.

### Data extraction and coding

For each RCT, we coded each medical practice being tested as novel or established at the time of the study. We defined established as being those practices having published proof of being used outside of clinical trials or were embodied in clinical guidelines that supported the use of the practice. We also coded articles as having positive, negative, or inconclusive results. A study was considered positive if it met its primary endpoint – significantly better for superiority trials and no worse for equivalence and noninferiority trials. Studies were coded as inconclusive if there was no clear benefit or harm from the intervention (e.g., progression free survival was better but quality of life was worse). Studies were considered negative if the study did not meet statistical significance in its primary endpoint. In studies that had both overall survival and progression-free survival as primary endpoints, overall survival was the outcome we used to determine whether the study met its endpoint.

### Data abstraction

For each final reversal study, we abstracted the funding source, year published, setting of the intervention, tumor type, and type of intervention. Funding source was categorized as industry only, non-industry only, or a combination of the two. The type of intervention was coded as medication, procedure, device, screening test, vitamins/supplements/food, behavioral therapy, radiation, or optimization. We considered optimization as a trial that compared different ways to administer an intervention (different doses, different orders). The intervention type “medication” was further categorized into cytotoxic, targeted, hormonal, radioactive, or other. We also created a variable that indicated roughly how long, at a minimum, the practice had been used outside of clinical trials. This documentation for all usage needed to come from the published scientific literature. We found the duration of use by abstracting either the date within the publication or the date of publication showing that the practice had been used (a more conservative estimate of duration of use) and finding the difference between this date and the date of reversal study publication. If the proof of evidence occurred during the same year as the study publication, the duration of the practice was coded as 1 year.

For all steps of study selection, two of four reviewers (D.H., A.H., T.C., J.G.) independently examined and abstracted information for each article. When there were differences in opinion between the two reviewers, adjudication first involved discussion between the two readers to see whether agreement could be reached. If disagreement persisted, a third reviewer (V.P.) adjudicated the discrepancy, as well as confirmed all potential reversals.

Studies that were both established and negative were included in our tentative list of oncology reversals. We used a two-step process to confirm the effectiveness of the practices on our tentative list of reversals [3]. In step one, we looked for a systematic review/meta-analysis to confirm or refute the results of the tentative reversal. Reviews were identified through PubMed suggestions when looking at the article on PubMed, and if no relevant review was found, we searched Google Scholar using search terms relating to the article. Reviews needed to include the tentative reversal study and the conclusions needed to be based on the RCT results only. Newer reviews were prioritized over older reviews. If a tentative reversal had a meta-analysis that refuted the results of its findings, it was removed from the reversal list. The final reversal list included articles that either did not have a systematic review to confirm the results or had a review that confirmed the results of the trial. For the second step, three practicing oncologists reviewed all reversals to confirm that they had been used and that the results were negative (V.P., E.Y.C., and T.H.). Our methods are similar to prior analyses [3–5].

### Data analysis

We calculated descriptive statistics on the final list of reversals in SAS software, version 9.4 (SAS Institute Inc., Cary, NC, USA). In accordance with 45 CFR §46.102(f), this study was not submitted for institutional review board approval because it involved publicly available data and did not involve individual patient data.

## Results

**Fig. 1:**
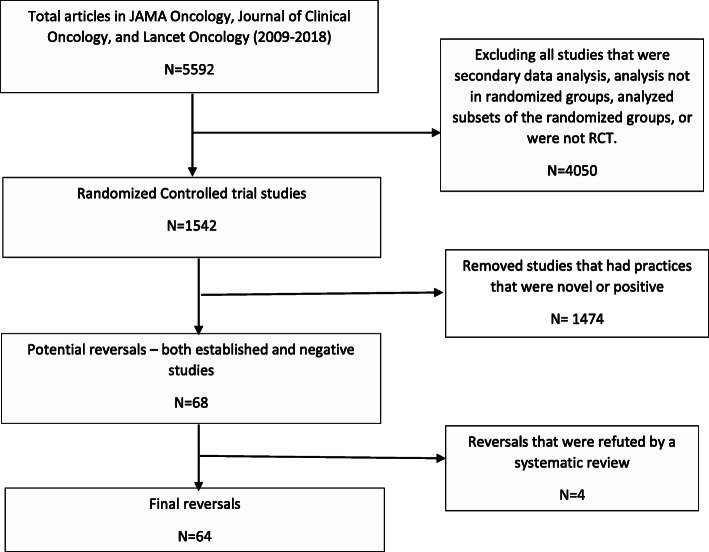
Flow diagram of the process of how oncology reversals were selected

There were 5592 total articles in high-impact oncology journals during the 10-year time frame. Of those, 1542 were randomized controlled trials. Of the RCTs, we found 781 (50%) studies that reached positive conclusions, 548 (36%) studies that were negative, and 213 (14%) that were inconclusive. There were 1047 (68%) studies pertaining to novel medical practices and 495 (32%) pertaining to established practices. We identified 68 potential reversals, i.e., practices for which RCTs were coded as negative pertaining to established interventions. The process for how studies were selected is depicted in Fig. 1.

Of the potential reversals, 20 (31%) were confirmed by a systematic review and four were refuted. Three (5%) practices had a meta-analysis that was inconclusive as to whether it supported the practice or not, and 41 (64%) did not have a meta-analysis done on that particular medical practice. Thus, our final list of oncology reversals included 64 studies (4% of all RCTs) [6–69]. These are all described in the Supplemental Table.

Thirty-two reversal studies (51%) were published in JCO, 28 (44%) were published in Lancet Oncology, and four (6%) were published in JAMA Oncology.

The breakdown of intervention type is as follows: 28 (44%) were medications, 10 (16%) were forms of radiotherapy or radiologic study, nine (14%) were procedures, six (9%) were optimization interventions, six (9%) were vitamins, supplements, or foods, four (6%) were behavioral therapies, and one (2%) was a screening test. For those that were medication interventions, 11 (39%) were targeted, six (21%) were cytotoxic, two (7%) were hormonal, none were radioactive, and nine (32%) were another type of medication.

The breakdown of tumor type is as follows: 18 (28%) were general/multiple, 13 (20%) were lung, 10 (16%) were breast, four (6%) were prostate, three (5%) were colorectal cancer, two (3%) were hepatocellular cancer, two (3%) were lymphoma, two (3%) were sarcoma, two (3%) were skin, two (3%) were endometrial, and six (10%) were another type of cancer.

As for funding, 14 (23%) were funded by pharmaceutical/industry only, 36 (59%) were funded by an organization other than pharmaceutical/industry, and 11 (18%) were a combination of industry and non-industry. Three studies either did not report funding or reported that there was no funding.

We found that the median number of years that the practice had been in use prior to the reversal study was 9 years (range 1–50 years). These characteristics are summarized in Table 1.

**Table 1:** Frequencies of characteristics of studies classified as medical reversals in Journal of American Medical Association (JAMA) Oncology, Journal of Clinical Oncology (JCO), and Lancet Oncology (2009–2018)

	JAMA Oncology (***N*** = 4)	JCO (***N*** = 32)	Lancet Oncology (***N*** = 28)	Combined (***N*** = 64)
**Minimum duration of time (years) that reversal was practiced, median (range)**	11·5 (9–17)	8 (1–37)	8 (1–50)	9 (1–50)
**Funding category, n (%)**				
**Industry**	1 (25)	8 (27)	5 (19)	14 (23)
**Non-industry**	3 (75)	15 (50)	18 (67)	36 (59)
**Combination of industry and non-industry**	0	7 (23)	4 (15)	11 (18)
**Not indicated**	0	1	2	3
**Reversal category, n (%)**				
**Medication**	1 (25)	17 (53)	10 (36)	28 (44)
**Radiation**	0	5 (16)	5 (18)	10 (16)
**Procedure**	1 (25)	1 (3)	7 (25)	9 (14)
**Optimization**	0	1 (3)	5 (18)	6 (9)
**Supplement/dietary**	2 (50)	4 (12)	0	6 (9)
**Behavioral**	0	4 (12)	0	4 (6)
**Screening test**	0	0	1 (4)	1 (2)
**Drug category, n (%)**				
**Targeted**	1 (100)	5 (29)	5 (50)	11 (39)
**Cytotoxic**	0	3 (18)	3 (30)	6 (21)
**Hormone**	0	1 (6)	1 (10)	2 (7)
**Other**	0	8 (47)	1 (10)	9 (32)
**Tumor type, n (%)**				
**General**	2 (50)	13 (41)	3 (11)	18 (28)
**Lung**	1 (25)	5 (16)	7 (25)	13 (20)
**Breast**	0	3 (9)	7 (25)	10 (16)
**Prostate**	0	2 (6)	2 (7)	4 (6)
**Colorectal**	0	1 (3)	2 (7)	3 (5)
**Hepatocellular**	0	1 (3)	1 (4)	2 (3)
**Endometrial**	0	2 (6)	0	2 (3)
**Lymphoma**	0	2 (6)	0	2 (3)
**Sarcoma**	0	0	2 (7)	2 (3)
**Skin**	0	0	2 (7)	2 (3)
**Other**	1 (25)	3 (9)	2 (7)	6 (9)

## Discussion

In our review of the three oncology journals the highest impact factor, we found 64 practices that have been used outside of clinical trials but were found to be ineffective in randomized trials. These practices represented the spectrum of cancer care, from prevention to screening to treatment (procedural or medication), and apply to all of the more common cancers. These practices constitute medical reversals. They were not replaced by better interventions; they were found to be no better than prior or lesser standards. In retrospect, their use failed to improve outcomes for patients. The implementation of practices prior to adequate evidence for their use is a central theme to the practices we identified. These practices are in addition to a list of medical reversals that we previously identified, which include oncology practices published on in general medical journals (e.g., New England Journal of Medicine, Lancet, and the Journal of the American Medical Association).

The reversals we found represented 4% of the oncology trials in the journals we examined. This percentage is similar to other studies that have found that 5% of oncology drugs receiving accelerated approval are withdrawn after post-marketing data are available [70]. These percentages are small and suggests that most of the practices in oncology that are being used have evidence backing their efficacy. The list of reversals that we have compiled shows the work that still needs to be done in identifying and eliminating medical reversals. As with medicine in general, a constant evaluation of the effectiveness of existing practices is central to the progress in oncology.

The identification of medical reversals or low-value practices is an important first step in eliminating their use. Their identification can then lead to clinical decision support tools and better communication between healthcare providers and patients, including educating patients about the value and effectiveness of medical practices, which have been identified as other important steps in reducing the usage of low-value practices [71]. The identification of these practices provides an opportunity for healthcare administrators, physicians and other healthcare providers to re-evaluate the care they provide, thus improving patient care and outcomes.

It is well acknowledged that the cost of cancer treatment is extremely high, and the financial toxicity is burdensome to patients and society at-large. Eliminating low-value care is key to easing the financial burden that comes with cancer treatment [72]. The identification of medical reversals, which are a subset of low-value practices, is one way to reduce health care costs through less money spent on ineffective practices. The Choosing Wisely campaign [73], which is one entity that lists low-value practices, has identified 75 practices in oncology (as of May 4, 2020), but this list is not comprehensive or objective, and relies on professional organizations to report which practices should be on the list. Adherence to Choosing Wisely recommendations has been estimated to be associated with a $19 million cost reduction per quarter for Medicare patients treated at cancer centers in the southwest region of the US [74]. The list we have assembled is objective and is comprehensive as far as what was published, and has the potential for notable savings if these practices were to be de-implemented.

An example of a practice failing to improve outcomes for patients is when first-generation EGFR inhibitors, such as erlotinib and gefitinib, were initially approved for 2nd and 3rd line metastatic non-small-cell lung cancer after a small improvement in survival endpoints. However, several later RCTs did not detect any survival advantage. It was not until additional studies were conducted that it was discovered that the subgroups with EGFR exon 19 and 21 abnormalities were the true responders [75]. Until the later studies, patients with non-EGFR mutations were exposed to costly drugs with potential side effects. This illustrates why we need to develop a good understanding of the molecular basis of novel therapies and identify the appropriate subgroups in an effort to prevent medical reversals from occurring.

Most reversals we found had little research previously published on them before the practices were implemented, as evident by the low percentage of practices with a systematic review done on them. However, a recent example of when a practice was implemented based on a randomized trial and then later reversed is olaratumab for patients with sarcoma. The accelerated approval by the US Food and Drug Administration in 2016 was based on a small phase II, randomized study, which led to thousands of patients receiving this drug [76]. But, when phase III trial data were released in 2019, olaratumab offered no overall survival benefit [77]. The drug has since been pulled from the market by the drug manufacturer. It is increasingly appreciated that small, underpowered studies that find benefits, particularly for outcomes that are not primary endpoints, often produce false positive or exaggerated results, which can lead to the widespread adoption of practices that provide no benefit [78].

We did examine the funding source of each of the reversals and found that most studies (77%) were funded by a non-industry organization, either in-part or in-full. Only 41% of the reversal studies we examined were in part industry funded. In contrast, 78% of randomized trials in oncology were funded in part by the industry over a comparable time period [79]. Industry funded trials are far more likely to be positive compared to trials funded by other entities and may be subject to methodological bias [80]. The lower numerical percentage of medical reversals that are funded by industry, in light of the much higher percentage of total clinical trials funded by industry, may be an indication of the pharmaceutical’s priority to fund novel interventions or promote the use of certain products. It is notable that 9 out of 11 (82%) medical reversals regarding targeted therapies are industry funded. This may reflect the high cost of novel agents preventing non-industry funded trials from evaluating these drugs. The burden of identifying practices that are ineffective or of low-value seems to fall more heavily on non-industry organizations. However, costly interventions may be prohibitively expensive to investigate without industry support. How to effectively direct public funding and motivate industry to generate high quality evidence is a complex issue warranting further research.

The lag time between use of a medical practice and subsequent reversal was found to be a median of 9 years in our analysis. This is the first reversal study to date that has sought to capture this lag [81, 82]. Notably our results are recapitulated in the FDA’s enforcement of post-marketing commitments for drugs receiving accelerated approval. These requirements are often delayed or incomplete [72, 83]. A lag between practice onset and definitive studies constitute an important period of time where the use of a practice, ultimately found ineffective or detrimental, was perpetuated without robust clinical trials. We believe it is important for the field to study strategies to minimize this time, and elsewhere have offered a number of solutions [84].

### Limitations

There are four limitations to our analysis. First, our study is not an exhaustive list of all oncology reversals. Several notable examples of medical reversals were not included on our list because the results were either published in other journals or have not been published to date. For example, olaratumab in combination with doxorubicin has been approved for use in patients with advanced or metastatic soft-tissue sarcoma, but to date, publication on the failure of the trial to show improvement in the primary endpoint of overall survival has only been published in abstract form or in non-scientific publications. With a reported 60% of negative trials (in either efficacy or safety) not being reported on in the literature [85], it is likely that our list is missing other noteworthy medical reversals.

Second, we only looked at three oncology journals, and, as such, our findings may not represent oncology practices at-large. Publication bias may also limit the generalizability. We chose these journals because they were more likely to publish studies on high-quality randomized trials. Third, the initial screening of each study was done by people without medical degrees in oncology, and some practices may not have been added to the list of reversals (possibly reducing sensitivity). Related to this is the limitation of subjectivity by different reviewers on whether practices were established, but notably each potential reversal was reviewed by three practicing medical oncologists. It is natural that others may disagree with our characterizations, and we encourage further independent research on this topic. Fourth, we were not able to capture the strength of recommendations when practices were implemented. Some of these recommendations were strong, while others were weak because of lack of definitive evidence. Finally, we recognize that not all of the identified medical reversals were widely implemented. We have included all practices that had documented use outside of clinical trials and case reports because sometimes when practices are used, even at low frequency, their use is perpetuated.

## Conclusion

In conclusion, we found that oncology reversals most often involve the administration of medications and are found in many oncology subspecialties. Further, we found that many of these reversals had been practiced for years and were often found to be a reversal because of studies funded predominately by non-industry organizations. Identification of medical reversals is an important step in being able to discontinue their use, which will help in directing resources towards practices that truly work. Our work also emphasizes the importance of robust governmental funding of cancer trials.

### Role of the funding source

The funder had no role in the design, analysis, or writing of this manuscript. The corresponding author had full access to all data used in this study and had final responsibility for the decision to submit for publication.

## Supplementary Information

**Additional file 1.**

## Data Availability

All data are publicly available.
