# Supplementary material for: The frequency of medical reversals in a cross-sectional analysis of high-impact oncology journals, 2009–2018
Source: BMC Cancer. 2021 Aug 3;21:889. doi: 10.1186/s12885-021-08632-8 (PMC8336285; doi:10.1186/s12885-021-08632-8)
Supplement: Supplementary file 1 — Additional file 1. [file 12885_2021_8632_MOESM1_ESM.docx]

**The frequency of medical reversals in a cross-sectional analysis of high-impact oncology journals, 2009-2018**

Alyson Haslam, PHD

Department of Epidemiology and Biostatistics, University of California, San Francisco

Jennifer Gill, MS

Providence Health & Services, Portland OR

Tyler Crain, MS

Department of Analytics, Northwest Permanente, Portland OR

Diana Herrera-Perez

Oregon Health & Science University, Portland OR

Emerson Y Chen, MD,

Oregon Health & Science University, Portland OR

Talal Hilal, MD

University of Mississippi Medical Center, Jackson MS

Myung S Kim, MD

Oregon Health & Science University, Portland OR

Vinay Prasad, MD, MPH*

Department of Epidemiology and Biostatistics, University of California, San Francisco

**Supplemental Table.** **Summary of studies classified as medical reversals in Journal of American Medical Association (JAMA) Oncology, Journal of Clinical Oncology (JCO), and Lancet Oncology (2009-2018).**

| **Author and Journal** | **Date** | **Title** | **Summary** | **funder** | **Reversal category** | **Duration of practice (minimum years)** |
| --- | --- | --- | --- | --- | --- | --- |
| Porceddu et al  JCO | 5/1/2018 | Postoperative Concurrent Chemoradiotherapy Versus Postoperative Radiotherapy in High-Risk Cutaneous Squamous Cell Carcinoma of the Head and Neck: The Randomized Phase III TROG 05.01 Trial | Concurrent, platinum-based, postoperative chemoradiotherapy has shown benefit in patients with high-risk mucosal squamous cell carcinoma of the head and neck (SCCHN).^1^ These results have been extrapolated to the treatment of cutaneous SCCHN, where retrospective review of patients charts have shown that high-risk patients have been treated with carboplatin in addition to concurrent chemoradiation.^2^ In a randomized trial where 157 patients were assigned to radiotherapy alone and 153 to concurrent chemotherapy in addition to postoperative radiotherapy, those assigned to concurrent chemotherapy did not have better freedom from locoregional relapse at either two (89% vs. 88%) or five years (87% vs 83%; p=0.58). This is a reversal of concurrent chemotherapy in addition to postoperative radiotherapy in patients with cutaneous SCCHN. | National Health and Medical Research Council–Australian Government, Cancer Council Queensland, and Queensland Government | Drug (cytotoxic) | 7 |
| Mok et al  JCO | 12/20/2017 | Gefitinib Plus Chemotherapy Versus Chemotherapy in Epidermal Growth Factor Receptor Mutation–Positive Non–Small-Cell Lung Cancer Resistant to First-Line Gefitinib (IMPRESS): Overall Survival and Biomarker Analyses | Epidermal growth factor receptor (EGFR) tyrosine kinase inhibitors are standard treatment for patients with non-small cell lung cancer (NSCLC) with an EGFR mutation. However, there has been a question as to whether or not to continue with an EGFR tyrosine kinase inhibitor after progression, in part because patients often become resistant to these drugs.^3^ Retrospective chart analysis shows variability in whether these treatments, including gefitinib, are offered after progression.^4,5^ Gefitinib is one of the options for treating these patients and was used in the IMPRESS randomized study on whether to continue with gefitinib in addition to chemotherapy (n=133) or treat with chemotherapy only (n=132). In the end, overall survival was lower in patients assigned gefitinib plus chemotherapy than in patients assigned chemotherapy only (HR=1.44; 95% CI=1.07-1.94). This is a reversal of continuing with gefitinib after progression in patients who have previously taken gefitinib for NSCLC. | AstraZeneca | Drug (targeted) | 7 |
| Chambers et al  JCO | 01/20/2017 | Mindfulness-Based Cognitive Therapy in Advanced Prostate Cancer: A Randomized Controlled Trial | Mindfulness-based cognitive therapy (MBCT) combines mindfulness-based stress reduction with cognitive behavioral therapy. It focuses on cognition and the relationship between thinking and mood, which may be beneficial for patients with cancer and whose rumination over their condition could lead to poorer health outcomes.^6^ MBCT has been used for almost a decade to minimize the psychological impact of cancer and treatment,^7^ and a book was published on this topic to specifically help cancer patients.^8^ In a randomized trial that assigned 94 men to a MBCT intervention and 95 men to minimally enhanced usual care, showed that MBCT was no better than usual care in reducing psychological distress (p=0.117), cancer specific distress (p=0.504), or prostate-specific antigen anxiety (p=0.220) in men with advanced prostate cancer . This is a reversal of the practice of MBCT for men with advanced prostate cancer. | Australian National Health and Medical Research grant | Behavioral | 10 |
| Demeestere et al  JCO | 08/01/2016 | No Evidence for the Benefit of Gonadotropin-Releasing Hormone Agonist in Preserving Ovarian Function and Fertility in Lymphoma Survivors Treated With Chemotherapy: Final Long-Term Report of a Prospective Randomized Trial | Chemotherapy and other cancer-related treatments at a young age can increase a woman's risk of infertility. Consequently, gonadotropin-releasing hormone agonists (GnRHa) have been used for several decades to prevent premature ovarian failure.^9^ GnRHa's are thought to preserve ovarian function because of their role in inducing a quiescent or ‘pre-pubertal' state in the ovaries, which would theoretically preserve ovarian function during chemotherapy.^10^ In a randomized study where 129 women (18-45 years of age) being treated for Hodgkin or non-Hodgkin lymphoma were assigned to either GnRHa therapy or no GnRHa therapy, neither ovarian reserve nor pregnancy (53% vs. 43% for GnRHa and control groups, respectively; p=.0467) had differences between the groups. This is a reversal for GnRHa in preserving ovarian function and fertility in women of child bearing age receiving treatment for Hodgkin or non-Hodgkin lymphoma. | Fonds National de la Recherche Scientifique (FNRS-Télévie) and by an unconditional grant from the Ipsen Pharmaceutical Group. Triptorelin was provided by the Ipsen Pharmaceutical Group. | Drug (hormone) | 20 |
| Brown et al  JCO | 06/10/2016 | Randomized Trial of Hepatic Artery Embolization for Hepatocellular Carcinoma Using Doxorubicin-Eluting Microspheres Compared With Embolization With Microspheres Alone | Transarterial chemoembolization (TACE) is standard of treatment for hepatocellular carcinoma, and has been shown to improve survival in these patients.^11^ In an effort to enhance the delivery of drugs to the tumor, drugs such as doxorubicin were added to the microspheres used in TACE. The belief of an added advantage with doxorubicin was confirmed in retrospective chart reviews comparing patients who underwent traditional TACE and those that received doxorubicin eluting beads.^12^ However, in a randomized trial of 51 patients who received microspheres alone and 50 patients who received doxorubicin beads, overall survival (20.8 vs. 19.6 months; p=0.64) was not any better with the addition of doxorubicin compared to microspheres alone. This is a reversal of doxorubicin-eluting beads for chemoembolization in patients with hepatocellular carcinoma. | NIH/NCI | Drug (cytotoxic) | 8 |
| van Hazel et al  JCO | 05/20/2016 | SIRFLOX: Randomized Phase III Trial Comparing First-Line mFOLFOX6 (Plus or Minus Bevacizumab) Versus mFOLFOX6 (Plus or Minus Bevacizumab) Plus Selective Internal Radiation Therapy in Patients With Metastatic Colorectal Cancer | Liver metastasis is the most common site of metastasis in colorectal cancer, and unfortunately, most individuals with liver metastasis are not candidates for surgery.^13^ Selective internal radiation therapy (SIRT) is a procedure that has been developed that helps deliver radiation directly into the hepatic artery, so that the tumor receives effective doses but normal tissue can be spared additional radiation.^14^ The technique is commercially available and was FDA approved in 2002,^14^ based on results from a smaller randomized trial.^15^ In a subsequent randomized study of 530 patients who received FOLFOX6, median progression free survival was no different in patients who received SIRT and those who did not (10.7 vs. 10.2 months; p=0.43). This is a reversal of the practice of SIRT for patients with colorectal cancer and liver metastasis. | Sirtex Technology | Radiation | 14 |
| Leyland-Jones et al  JCO | 04/10/2016 | A Randomized, Open-Label, Multicenter, Phase III Study of Epoetin Alfa Versus Best Standard of Care in Anemic Patients With Metastatic Breast Cancer Receiving Standard Chemotherapy | Erythropoietin-stimulating agents are often prescribed to patients who are undergoing chemotherapy in order to treat anemia, which is often a side-effect of chemotherapy regimens. In one survey, it was estimated that over 60% of physicians inside or outside of the US prescribe erythropoietin to patients with hemoglobin concentrations equal to or less than 11 g/dL who have breast cancer and are treated with chemotherapy.^16^ Because of safety concerns regarding the use of erythropoietin in oncology patients, a noninferiority randomized trial was performed. This trial that assigned 1,050 to erythropoietin and 1,048 to standard care, reported that erythropoietin failed to be equivalent to standard of care in preventing deaths. There was a 9% higher death rate in the erythropoietin group. This is a reversal of the practice of erythropoietin administration to patients undergoing chemotherapy. | Janssen Research & Development | Drug (other) | 2 |
| Fallon et al  JCO | 02/20/2016 | Randomized Double-Blind Trial of Pregabalin Versus Placebo in Conjunction With Palliative Radiotherapy for Cancer-Induced Bone Pain | Bone pain is a common occurrence for patients with cancer. Radiotherapy and morphine are the main treatment options for treating pain in patients with cancer, but pregabalin is being used more commonly, even though there is little evidence to support its use.^17^ In a randomized trial that included 233 patients who were treated with radiotherapy, the addition of pregabalin did not lead to better treatment responses (reduction of >1 points in worst pain by week 4) compared to placebo (38.8% vs. 40.2%; p=0.816). This is a reversal for the use of pregabalin in addition to radiotherapy in cancer-induced bone pain. | Cancer Research UK Grants No. A7250 and A11922 and educational grant A7250 from Pfizer UK | Drug (other) | 4 |
| Nicolajie et al  JCO | 11/01/2015 | Impact of an Automatically Generated Cancer Survivorship Care Plan on Patient-Reported Outcomes in Routine Clinical Practice: Longitudinal Outcomes of a Pragmatic, Cluster Randomized Trial | Survivorship care plans (SCP) were developed as a way for patients and physicians to monitor document treatment and follow-up plans for the patient and to facilitate communication,^18^ and in 2006, The Institute of Medicine recommended that all cancer patients receive a SCP.^19^ In a randomized trial of 221 patients, patients assigned to SCP did not have better scores in regards to satisfaction with care (71 vs 68; difference=4.5; 95% CI=-2.4 to 11.3) compared to patients who received usual care, and those with SCPs had more symptoms (3.3 vs.2.6; p=0.03), were more concerned about their illness (4.4 vs. 2.3; p=0.03), and were more affected emotionally (4.0 vs. 3.7; p=0.046). This is a reversal of the practice of routine use of SCPs in oncology. | Dutch Cancer Society | Behavioral | 9 |
| Pivot et al  JCO | 05/10/2015 | CEREBEL (EGF111438): A Phase III, Randomized, Open-Label Study of Lapatinib Plus Capecitabine Versus Trastuzumab Plus Capecitabine in Patients With Human Epidermal Growth Factor Receptor 2–Positive Metastatic Breast Cancer | Brain metastasis can sometimes occur because traditional treatments, including trastuzumab, do not cross the blood-brain barrier, where cancer cells may have spread.^20^ Lapatinib has shown the potential to cross the blood-brain barrier, and therefore may better suited to treat breast cancer patients with brain metastasis. Lapatinib in combination with capecitabine was approved in 2007 as a second line therapy by the FDA for patients with HER2-positive metastatic breast cancer,^21^ and was granted conditional approval in 2008 by the European Medicines Agency.^22^ Further, results of a phase-II single-arm study encouraged the use of lapatinib plus capecitabine for this indication.^23^ In the CEREBEL trial, 540 patients with HER2-positive breast cancer and central nervous system (CNS) metastases were randomized to either a lapatinib-capecitabine combination or a trastuzumab-capecitabine combination. In this trial, CNS metastasis was no better in the lapatinib-capecitabine group (3% vs. 5%; p=0.360), and overall survival (HR=1.34; 95% CI=0.95 to 1.64) and progression-free survival (HR=1.30; 95% CI=1.04 to 1.64) were worse. This is a reversal for lapatinib plus capecitabine for treating patients with metastatic HER2-positive breast cancer and CNS metastasis. | GlaxoSmithKline | Drug (targeted) | 8 |
| Paulson et al  JCO | 10/10/2014 | Efficacy of Methylprednisolone on Pain, Fatigue, and Appetite Loss in Patients With Advanced Cancer Using Opioids: A Randomized, Placebo-Controlled, Double-Blind Trial | Several methods are used to control pain associated with cancer, which is common. Opioids are a commonly used method but corticosteroids are commonly used in conjunction with opioids to control pain and possibly reduce opioid needs,^24^ and the combination is part of cancer pain guidelines.^25^ In a randomized study, 592 patients being treated with opioids for cancer were assigned either methylprednisolone or placebo. In the end, there were no differences in pain between the two groups (1.19 vs. 1.20, for methylprednisolone and placebo, respectively; p=0.88). This is a reversal of methylprednisolone to augment opioids in pain management for patients with cancer. | Telemark Hospital Trust and the South-Eastern Norway Regional Health Authority | Drug (other) | 7 |
| Fernando et al  JCO | 08/10/2014 | Impact of Brachytherapy on Local Recurrence Rates After Sublobar Resection: Results From ACOSOG Z4032 (Alliance), a Phase III Randomized Trial for High-Risk Operable Non–Small-Cell Lung Cancer | Sublobular resection is a common alternative to lobectomy in patients with stage I non-small cell lung cancer (NSCLC), especially in people with compromised cardiopulmonary reserve. The downside to this procedure is that there is a high recurrence rate. The use of brachytherapy with Iodine-125 was first reported on in 1998, as it was thought that this practice delivered concentrated radiation to the resection margin with less of an impact on surrounding tissue.^26^ Since then, this practice has been reported in multiple observational and retrospective studies at multiple institutions.^27^ In a randomized controlled trial of 224 patients, sublobular resection with brachiotherapy did not lead to better local recurrence when compared to sublobular resection only (HR=1.01; 95% CI=0.51 to 1.98; p=0.98). This is a reversal of the practice of brachiotherapy for patients with stage I NSCLC receiving sublobular resection. | National Cancer Institute (NCI) U10 Grant No. CA076001 and by an additional grant from Oncura, | Procedure | 16 |
| Kawaguchi et al  JCO | 06/20/2014 | Randomized Phase III Trial of Erlotinib Versus Docetaxel As Second- or Third-Line Therapy in Patients With Advanced Non–Small-Cell Lung Cancer: Docetaxel and Erlotinib Lung Cancer Trial (DELTA) | Non-small cell lung cancer (NSCLC) has a poor prognosis, and patients are often put on multiple lines of therapy because of failure after a platinum-based first line treatment. Subsequent therapy can include docetaxel and erlotinib.^28^ Erlotnib was approved in 2005 in Canada as a second line or later monotherapy in patients with NSCLC,^28^ but retrospective review of patient charts show this medical practice to be used even before then.^28^ In the DELTA randomized trial of unselected patients, including 150 patients with NSCLC treated with erlotinib and 151 patients treated with docetaxel in the second or third line, there was no difference in median overall survival between those treated with erlotinib and those treated with docetaxel (14.8 months vs. 12.2 months; p=0.53). This is a reversal of the practice of erlotinib in second or third line treatment of NSCLC in unselected patients. | Japanese National Hospital Organization Multi-Center Clinical Research for Evidence-Based Medicine, Japan | Drug (targeted) | 6 |
| Smith et al  JCO | 04/10/2014 | Randomized Controlled Trial of Early Zoledronic Acid in Men With Castration-Sensitive Prostate Cancer and Bone Metastases: Results of CALGB 90202 (Alliance) | Bone metastases can be a serious outcome in men with prostate cancer. Zoledronic acid is thought to be helpful for patients with bone metastases because it inhibits bone resorption, thus minimizing osteoclast-mediated bone destruction that often accompanies bone metastases and prevents bone loss due to other hormonal treatments.^29,30^ The use of zolendronic acid in conjunction with hormone therapy has been recommended by a panel of experts that convened 3rd International Consultation  on Prostate Cancer.^31^ One trial randomized 645 men with castration-sensitive prostate cancer and at least one bone metastases to either zolendronic acid or placebo. The median time to first skeletal-related event was no different between those given zolendronic acid and those given placebo (31.9 months; 95% CI, 24.2 to 40.3 vs. 29.8 months; 95% CI, 25.3 to 37.2; hazard ratio, 0.97; 95% CI, 0 to 1.17; one-sided stratified log-rank P = .39). Further, overall survival was no different (hazard ratio, 0.88; 95% CI, 0.70 to 1.12; p = 0.29). This is a reversal of zolendroic acid to prevent skeletal-related events in men with castration sensitive prostate cancer and bone metastases. | National Cancer Institute to the Alliance for Clinical Trials in Oncology and to the Alliance Statistics and Data Center, as well as Novartis Oncology, and research awards from the Prostate Cancer Foundation | Drug (other) | 12 |
| Hoff et al  JCO | 04/01/2014 | Randomized Phase III Trial Exploring the Use of Long-Acting Release Octreotide in the Prevention of Chemotherapy-Induced Diarrhea in Patients With Colorectal Cancer: The LARCID Trial | Diarrhea is a common side-effect from chemotherapy treatment. Octreotide is a drug used for this condition because of its ability to inhibit gastrointestinal motility, pancreatic secretion, and intestinal absorption.^32^ Several organizations and groups have recommend this as treatment, and at least one goes so far as recommending a long-acting formulation for people who do not desire a dose reduction.^33,34^ In the LARCID trial, 139 patients with colorectal cancer and chemotherapy-induced diarrhea were randomly assigned to either long-acting octreotide or physician's choice of treatment. The rate of diarrhea was no different between the octreotide arm and the physician's choice arm (76.1% vs. 78.9%; p>0.05). This is a reversal of the practice of long-acting octreotide for preventing diarrhea in patients with colorectal cancer and chemotherapy-induced diarrhea. | Mitsuhiro Kamimura, Chugai Pharmaceutical; Hideo Saka, Chugai Pharmaceutical | Drug (other) | 7 |
| Loprinzi et al  JCO | 04/01/2014 | Phase III Randomized, Placebo-Controlled, Double-Blind Study of Intravenous Calcium and Magnesium to Prevent Oxaliplatin-Induced Sensory Neurotoxicity (N08CB/Alliance) | For patients receiving FOLFOX (fluorouracil, leucovorin, and oxaliplatin) therapy for cancer treatment, neuropathy is a common side-effect that can last for several years.^35^ Because calcium and magnesium are involved in nervous system polarization and the hyperexcitability of neurons from chelation of calcium by oxalates, the combination of the two minerals was believed to provide benefit in patients who experience neuropathy.^36^ Some medical facilities began using calcium and magnesium infusions on patients receiving oxaliplatin chemotherapy regimens, and retrospective analysis of patient data, showed a possible benefit for this practice.^37^ One survey of Australian physicians indicated that over one-third (34.6%) of respondents reported using calcium and magnesium to prevent neurotoxicity from oxaliplatin-based chemotherapy.^38^ In a randomized trial of 353 patients with colon cancer undergoing adjuvant therapy with FOLFOX, there was some indication that calcium and magnesium reduced discomfort from swallowing cold liquids, there were no differences in sensitivities to touching cold items (p=0.04), throat discomfort, or muscle cramps between those receiving calcium and magnesium and those receiving placebo (p=0.80, 0.43, and 0.55, respectively). This is a reversal of calcium and magnesium for preventing FOLFOX-related neuropathy. | Public Health Service | Drug (other) | 8 |
| Onsrud et al  JCO | 11/1/2013 | Long-Term Outcomes After Pelvic Radiation for Early-Stage Endometrial Cancer | Stage-I endometrial cancer is very treatable and has a high survival rate, but because relapse can occur there has been differing views in how to prevent relapses.^39^ Surgery is the most commonly performed treatment for stage-I endometrial cancer, but brachytherapy and postoperative external beam radiation therapy (EBRT) after surgery have sometimes been used as a means of reducing recurrence.^40,41^ After 20 years of follow-up, there was no difference in survival (p=0.186), and in those younger than 60 years of age, mortality rates (HR, 1.36; 95% CI, 1.06 to 1.76) and secondary cancer occurrence (HR, 2.02; 95% CI, 1.30 to 3.15) were who receive EBRT. This is a reversal of postoperative EBRT in patients with stage-I endometrial cancer. | not indicated | Radiation | 37 |
| Hollingworth et al  JCO | 10/10/2013 | Are Needs Assessments Cost Effective in Reducing Distress Among Patients With Cancer? A Randomized Controlled Trial Using the Distress Thermometer and Problem List | Cancer, in addition to being a physical disease, can also place a high emotional toll on an individual. Numerous methods have been developed to help physicians and caregivers assess distress among cancer patients, including the Distress Thermometer and Problem List (DT&PL), which is widely used.^42,43^ One randomized trial where 220 patients with a primary tumor were assigned to DT&PL or usual care, there was no difference in profile of mood states between the groups (difference between groups, −1.84; 95% CI, −5.69 to 2.01; p=0.35). This is a reversal of DT&PL to assess distress in patients with cancer. | National Institute for Health Research, Research for Patient Benefit | Optimization | 1 |
| Gordon et al  JCO | 02/20/2013 | Randomized Phase III Trial of ABVD Versus Stanford V With or Without Radiation Therapy in Locally Extensive and Advanced-Stage Hodgkin Lymphoma: An Intergroup Study Coordinated by the Eastern Cooperative Oncology Group (E2496) | Doxorubicin, bleomycin, vinblastine, and dacarbazine (ABVD) has been considered standard of care for patients with advanced Hodgkin's lymphoma for several decades. The Stanford V regimen was introduced as a treatment option in the 1990s for those with advanced disease after single arm trials showed this regimen showed favorable results.^44,45^ However, this regimen was introduced before results of randomized trials were conducted to show its superiority. Later, a trial randomized 428 patients with advanced Hodgkin's to ABVD and 426 patients to the Stanford V regimen. In the end there were no differences in failure free survival at 5 years between the two groups 74% for ABVD and 71% for Stanford V (p=0.32), and in the Stanford V arm, compared with the ABVD arm, there were more instances of grade 3 lymphopenia (p<0.001), grade 3 or 4 leukocytopenia (p<0.001), grades 3 and 4 sensory neuropathy (p<0.001), and grade 3 or 4 motor neuropathy (p=0.006) This is a reversal of the Stanford V regimen for patients with advance Hodgkin's lymphoma. | Public Health Service, National Cancer Institute, National Institutes of Health | Drug (cytotoxic) | 17 |
| Soffietti et al  JCO | 01/01/2013 | A European Organisation for Research and Treatment of Cancer Phase III Trial of Adjuvant Whole-Brain Radiotherapy Versus Observation in Patients With One to Three Brain Metastases From Solid Tumors After Surgical Resection or Radiosurgery: Quality-of-Life Results | Stereotactic radiosurgery is standard of care for patients who have brain metastases from a solid tumor. Whole brain radiotherapy (WBRT) was first introduced in the 1950s as an adjuvant treatment for these patients.^46^ It became widely used after studies found that it improved cognitive outcomes even though there was little evidence showing an improvement in overall survival or quality of life.^47^ A randomized trial of 359 patients with solid tumors and brain metastases showed no improvement in quality of life, and in fact, was detrimental on quality of life in patients who were assigned to WBRT, compared to observation (mean, 63.2; SE, 3.2 for observation v mean, 52.2; SE, 3.2 for WBRT; p=0.0148). This is a reversal of adjuvant WBRT for patients with brain metastases from solid tumors who undergo stereotactic radiosurgery. | Fonds Cancer from Belgium | Radiation | 1 |
| Bruera et al  JCO | 01/01/2013 | Parenteral Hydration in Patients With Advanced Cancer: A Multicenter, Double-Blind, Placebo-Controlled Randomized Trial | Parenteral hydration at the end of life for patients with advanced cancer and who are in hospice can be a complex issue because of the need to weigh the benefits and risks of this practice. Patients will often naturally decrease their intake of fluids but this can exacerbate their symptoms.^48^ And, because there are not hard and fast guidelines, this practice is sometimes followed and sometimes not.^48^ Some facilities and caregivers do not routinely provide parenteral hydration, but will at the request of family members.^49^ In a randomized trial where patients were given either parenteral hydration (n=63) or placebo (n=66), dehydration symptoms (−3.3 v −2.8, p=0.77), Edmonton Symptom Assessment Scale (all nonsignificant), Memorial Delirium Assessment Scale (1 v 3.5, p=0.084), Nursing Delirium Screening Scale (0 v 0, p=0.13), and Unified Myoclonus Rating Scale (0 v 0, p=0.54) and overall survival (median, 21 v 15 days, p=0.83) were no different between the groups. This is a reversal of parenteral hydration for patients who had advanced care and who were in hospice care. | National Institutes of Health, the MD Anderson Cancer Center Support Grant, an institutional startup fund | Supplement/dietary | 18 |
| Cruciani et al  JCO | 11/01/2012 | L-Carnitine Supplementation for the Management of Fatigue in Patients With Cancer: An Eastern Cooperative Oncology Group Phase III, Randomized, Double-Blind, Placebo-Controlled Trial | Since fatigue is common for patients with cancer, many interventions have been prescribed - either via doctor’s orders or self-prescription. L-carnitine, an over-the-counter supplement, is a popular choice because of its involvement with metabolism and possibly upregulating the energy production in those with L-carnitine deficiency, which is common in people with chronic conditions such as cancer.^50^ Integrative physicians have made recommendations for dose and duration for this supplement as a way to reduce fatigue in patients with cancer and other chronic conditions.^51^ In a trial where 376 patients were randomly assigned to either carnitine or placebo, there was an improvement in fatigue symptoms in both treatment arms (L-carnitine: −0.96, 95% CI, −1.32 to −0.60; placebo: −1.11, 95% CI −1.44 to −0.78), but neither treatment improved fatigue more than the other (p=0.57). This is a reversal of L-carnitine supplementation for fatigue in patients with cancer. | National Cancer Institute | Supplement/dietary | 1 |
| Grunfeld et al  JCO | 12/20/2011 | Evaluating Survivorship Care Plans: Results of a Randomized, Clinical Trial of Patients With Breast Cancer | In 2006, the Institute of Medicine published a report on health care for cancer survivors in which it recommended that patients who completed primary care should be provided with a survivorship care plan (SCP).^52^ The SCP was meant to offer support, information, guidelines, and recommendations on how to navigate life as a cancer survivor.^53^ SCPs have been widely adopted,^54^ yet the effectiveness of the program had not been rigorously studied. This randomized trial sought to evaluate the effect of SCPs on patient-reported outcomes in women with early-stage breast cancer who completed primary treatment. The investigators found no difference in cancer-related distress, quality of life, or patient satisfaction when comparing patients who received a SCP at discharge and those who did not. This is a reversal of creating and distributing survivorship care plans for breast cancer patients who have completed primary treatment. | Canadian Breast Cancer Research Alliance, and the Ontario Institute for Cancer Research with funds from the Ontario Ministry of Research and Innovation | Behavioral | 3 |
| Blohmer et al  JCO | 10/01/2011 | Randomized Phase III Trial of Sequential Adjuvant Chemoradiotherapy With or Without Erythropoietin Alfa in Patients With High-Risk Cervical Cancer: Results of the NOGGO-AGO Intergroup Study | Anemia is associated with a worse prognosis for cancer patients,^55^ and therefore treating anemia was explored as an option for improving health outcomes in cancer patients. Erythropoietin alfa (EPO) is an agent shown to increase hemoglobin concentrations and reduce the need for blood transfusions in cancer patients,^56^ and has therefore been administered to cancer patients to improve clinical outcomes.^57^ This randomized study investigated the effects of EPO with adjuvant chemotherapy and pelvic radiotherapy in cervical cancer patients who had undergone radical hysterectomy. The investigators found that, when comparing adjuvant therapy plus EPO to adjuvant therapy alone, there was no difference in recurrence-free survival (HR, 0.66; 95% CI, 0.39-1.12; p=0.06) or in overall survival (HR, 0.88; 95% CI, 0.51-1.50; p=0.63). This is a reversal of adding EPO to adjuvant chemoradiotherapy in patients with stage IB to II cervical cancer who had undergone radical hysterectomy. | Humboldt-University Berlin, Ortho Biotech Germany (division of Janssen-Cilag), Amgen Germany, and ASTA Pharmaceuticals Germany | Drug (other) | 10 |
| Nout et al  JCO | 05/01/2011 | Long-Term Outcome and Quality of Life of Patients With Endometrial Carcinoma Treated With or Without Pelvic Radiotherapy in the Post Operative Radiation Therapy in Endometrial Carcinoma 1 (PORTEC-1) Trial | In the 1990s, pelvic external beam radiotherapy (EBRT) was a common postoperative treatment for patients with endometrial carcinoma of any stage.^58^ The Post Operative Radiation Therapy in Endometrial Carcinoma 1 (PORTEC-1) trial determined that EBRT did not improve overall survival and led to an increased risk of adverse effects in patients with stage-1 endometrial carcinoma.^59^ This follow up trial of PORTEC-1 sought to determine the long-term effects of pelvic EBRT on survival and quality of life and found similar results. Overall survival was 52% in the EBRT group versus 60% in the surgery alone group (HR, 0.84; 95%CI, 0.67-1.06; p=0.14). The EBRT group had higher rates of urinary incontinence, diarrhea, and fecal leakage, and reported that they were more limited in daily activities (p< 0.01). The EBRT group also had lower physical functioning scores compared to the surgery alone group (p=0.004). This is a reversal of postoperative pelvic EBRT for patients with stage 1 endometrial carcinoma. | Dutch Cancer Society | Drug (other) | 10 |
| Kocher et al  JCO | 01/10/2011 | Adjuvant Whole-Brain Radiotherapy Versus Observation After Radiosurgery or Surgical Resection of One to Three Cerebral Metastases: Results of the EORTC 22952-26001 Study | Whole-brain radiotherapy (WBRT) was commonly used as an adjuvant treatment after surgery or radiosurgery for patients with brain metastases.^60^ While WBRT was thought to reduce tumor recurrence and protect neurocognitive function,^61^ the treatment was associated with neurotoxicities and was not shown to improve overall survival. In this trial, the European Organisation for Research and Treatment of Cancer compared the effects of WBRT (n=180) versus observation (n=179) in patients with brain metastases from solid tumors (excluding small-cell lung cancer) who were treated with complete surgery or radiosurgery. Median performance status deterioration was 10.0 months after observation and 9.5 months after WBRT (HR, 0.96; 95%CI, 0.76-1.20; p=0.71). Overall survival was also not different between the groups (10.9 months observation v 10.7 months WBRT; p=0.89). Although WBRT reduced intracranial relapses and neurological deaths, it did not improve duration of function independence or overall survival. This is a reversal of adjuvant WBRT after radiosurgery or surgical resection for patients with one to three cerebral metastases. | National Cancer Institute (Bethesda, MD) and by a donation from the Deutsche Krebshilfe from Germany through the EORTC Charitable Trust | Radiation | 12 |
| Steensma et al  JCO | 01/01/2011 | Phase III, Randomized Study of the Effects of Parenteral Iron, Oral Iron, or No Iron Supplementation on the Erythropoietic Response to Darbepoetin Alfa for Patients With Chemotherapy-Associated Anemia | Erythropoiesis-stimulating agents (ESAs) are used to treat chemotherapy-associated anemia, increasing hemoglobin levels and reducing the need for blood transfusions in many cancer patients.^62^ Yet there are patients who do not respond to ESA treatment, many times due to iron deficiency. Guidelines on chemo-associated anemia are vague in their recommendations, offering iron supplementation as a treatment option alongside ESA treatment, both oral and parenteral, while warning that data are limited on their efficacy in this setting.^63,64^ This study compared the effects of intravenous ferric gluconate (n=167), oral ferrous sulfate (n=168), and oral placebo (n=167) in patients with hemoglobin less than 11 g/dL undergoing chemotherapy and receiving darbepoetin. Neither IV nor oral iron improved erythropoietic response rate compared to placebo (IV iron, 69.5% vs oral iron, 66.9% vs oral placebo 65.0%; p=0.75). Rates of red cell transfusions and quality of life changes were also similar in all groups. This is a reversal of adding IV ferric gluconate or oral ferrous sulfate to darbepoetin in patients with chemotherapy-associated anemia. | Public Health Service Grant and a grant from Amgen to the Mayo Clinic Cancer Research Consortium | Supplement/dietary | 4 |
| Wolf et al  JCO | 12/10/2010 | Placebo-Controlled Trial to Determine the Effectiveness of a Urea/Lactic Acid–Based Topical Keratolytic Agent for Prevention of Capecitabine-Induced Hand-Foot Syndrome: North Central Cancer Treatment Group Study N05C5 | Hand-foot syndrome caused by certain chemotherapy agents can be painful and reduce quality of life in patients with cancer.^65^ Cotaryl cream, which contains urea and lactic acid, is a topical ointment with keratolytix and hydrating properties that is often used to treat skin conditions such as eczema and xerosis.^66^ Cotaryl cream was also shown to improve symptoms of hand-foot syndrome in a pilot study, and anecdotal evidence has supported it use.^67,68^ This study randomized patients receiving capecitabine to urea/lactic acid-based topical keratolyric agent (ULABTKA, n=70) or placebo cream (n=67) and found that the incidence of moderate/severe patient-reported hand-foot symptoms did not differ between groups (13.6% in the ULABTKA arm vs 10.2% in the placebo arm; p=0.768). This is a reversal of ULABTKA for the prevention of capecitbaine-induced hand-foot syndrome. | National Cancer Institute Community Clinical Oncology Program Grant, by the National Institutes of Health, and by Roche Pharmaceuticals | Drug (other) | 2 |
| Kang et al  JCO | 08/20/2010 | Pyridoxine Is Not Effective to Prevent Hand-Foot Syndrome Associated With Capecitabine Therapy: Results of a Randomized, Double-Blind, Placebo-Controlled Study | Hand-foot syndrome (HFS), or palmar-plantar erythrodysesthesia, is a painful dermatological reaction that often manifests as an adverse effect of capecitabine-containing chemotherapy.^69^ HFS has similar symptoms to acrodynia, a disease caused by pyridoxine deficiency, therefore HFS was sometimes treated with pyridoxine, a form of vitamin B6.^70^ This study compared HFS occurrence in patients receiving oral pyridoxine (n=180) and patients receiving placebo (n=180) and found that pyridoxine was not effective at preventing capecitabine-associated HFS. The cumulative dose of capecitabine until grade 2 or worse HFS did not differ between group (HR, 0.95; p=0.788). This is a reversal of pyridoxine for preventing capecitabine associated HFS. | not fully stated but Samil Pharmaceuticals provided both pyridoxine and the placebo for this trial | Supplement/dietary | 8 |
| Hauschild et al  JCO | 07/20/2009 | Prospective Randomized Multicenter Adjuvant Dermatologic Cooperative Oncology Group Trial of Low-Dose Interferon Alfa-2b With or Without a Modified High-Dose Interferon Alfa-2b Induction Phase in Patients With Lymph Node–Negative Melanoma | In patients with high-risk cutaneous melanoma, adjuvant treatment with interferon alpha 2b (IFN-a2b) has shown clinical benefit after surgery and was adopted into practice.^71^ IFN-a2b was administered in both high and low intensity regimens,^72^ although there was no consensus as to whether high-dose schedules were more beneficial.^73^ This randomized study compared the effects of high-dose IFN-a2b (n=321) to low-dose IFN-a2b (n=329) in lymph node-negative patients with resected primary malignant melanoma of more than 1.5-mm tumor thickness. Five-year relapse-free survival rates were 68.0% in the high-dose group and 67.1% in the low dose group (p=0.90). Similarly, 5-year overall survival was not statistically significant at 77.8% versus 79.8% (p=0.68). This is a reversal of high-dose IFN-a2b induction in patients with lymph node-negative melanoma. | Schering-Plough | Drug (targeted) | ? |
| Goss et al  JCO | 05/01/2009 | Randomized Phase II Study of Gefitinib Compared With Placebo in Chemotherapy-Naive Patients With Advanced Non–Small-Cell Lung Cancer and Poor Performance Status | Patients with non-small cell lung cancer (NSCLC) and poor performance status (PPS) often have limited treatment options because they often have unfavorable responses to traditionally recommended first-line chemotherapy. As such, there are no standard treatment recommendations and physicians are left to choose from a list of options with few data supporting their use in this population.^74^ Gefitinib is one treatment option that was approved in 2001 by the FDA and several other regulatory bodies for the first-line treatment of NSCLC, ^74^ and it is recommended by a European consensus panel as a first line treatment in patients with NSCLC and PPS.^75^ In a randomized trial, 100 patients with advanced NSCLC and PPS were assigned to gefitinib and 101 patients assigned to placebo. Patients assigned to gefitinib had no better progression free survival (0.82; 95% CI, 0.60 to 1.12; p=0.217) or overall survival (0.84; 95% CI, 0.62 to 1.15; p=0.272) than patients assigned to placebo. This is a reversal of first-line gefinib for patients with advanced NSCLC and PSS. | AstraZeneca | Drug (targeted) | 8 |
| Mills et al  JCO | 01/01/2009 | Does a Patient-Held Quality-of-Life Diary Benefit Patients With Inoperable Lung Cancer? | Measuring quality-of-life (QOL) is important in palliative care settings, yet there is no consensus on how to apply QOL measurements in clinical practices, resulting in a slow to adoption of routine QOL measurement in clinical practice. In their guidelines for end-of-life care in patients with lung cancer, the American College of Chest Physicians recommends that QOL measurements be routinely administered by practitioners to improve patient care.^76^ This trial randomized patients with inoperable lung cancer to receive standard care (n=58) or a structured QOL diary (n=57) and found that the diary group fared worse than the standard care group. QOL was insignificantly but consistency worse in the diary group compared to the standard group and there were no differences in satisfaction with care, communication, or discussion of patient problems between groups. The authors conclude that QOL questionnaires without appropriate support and feedback for patients and health care professionals may have negative impacts on patients in palliative care. This is a reversal of patient-held QOL diaries for patients with inoperable lung cancer. | Northern Ireland Research and Development Office | Behavioral | 2 |
| Galimberti et al  Lancet Oncology | Oct 2018 | Axillary dissection versus no axillary dissection in patients with breast cancer and sentinel-node micrometastases (IBCSG 23-01): 10-year follow-up of a randomised, controlled phase 3 trial | Prior to the year 2000, most women with breast cancer and micrometastases had complete axillary lymph node dissection (ALND) as part of their surgical treatment^77^, The development of radiocolloid lymphatic tracers and dyes in the 1990s reduced the need for routine ALND in these patients because of the improved ability to detect whether or not there was micrometastases.^78^ In a randomized trial of 6681 patients with breast cancer and one or more positive sentinel nodes (largest lesion size of 5 cm), neither overall survival (HR=0.78; 95% CI=0.53 to 1.14; p=0.20) nor disease-free survival (HR=0.85; 95% CI=0.65 to 1.11; p=0.24) were any different between those with no ALND and those with ALND. This is a reversal of routine ALND for patients with breast cancer (largest lesion size of 5 cm). | International Breast Cancer Study Group | Procedure | 18 |
| Negenborn et al  Lancet Oncology | Sep 2018 | Quality of life and patient satisfaction after one-stage implant-based breast reconstruction with an acellular dermal matrix versus two-stage breast reconstruction (BRIOS): primary outcome of a randomised, controlled trial | Implant-based breast reconstruction (IBBR) is commonly performed after a mastectomy, and several methods have been performed. The development of acellular dermal matrices (ADMs) has allowed breast reconstruction to be done at the time of the mastectomy surgery (one-stage) instead of in a subsequent surgery.^79^ The convenience of this technology has led to almost 85% of US surgeons using ADMs, even though there is a high financial cost associated with ADMS and a high degree of skill to place them.^80^ A recent randomized trial assigned 69 women to IBBR with ADMs and 73 to a two-stage IBBR without ADMs. This study found that there were no better aesthetic outcomes between the two groups (breast satisfaction: 80% vs. 70.5; p=0.35), and the women assigned to one-step IBBR with ADMs had a higher frequency of surgical complications and reoperations (29% vs. 7%; p=not reported). This is a reversal of one-stage IBBR with ADMs in women having breast reconstruction after mastectomy. | Pink Ribbon, Nuts-Ohra, and LifeCell | Optimization | 4 |
| van den Bent et al  Lancet Oncology | Sep 2018 | Bevacizumab and temozolomide in patients with first recurrence of WHO grade II and III glioma, without 1p/19q co-deletion (TAVAREC): a randomised controlled phase 2 EORTC trial | Patients with grade II and II glioma have a better prognosis than glioblastoma, but relapse is common.^81^ Bevacizumab is FDA approved for several cancers, including glioblastoma, and has been used in off-label settings for the treatment of individuals with grade II and III gliomas.^82,83^ In a randomized trial of 155 patients, patients with grade II and III glioma recurrence who were assigned bevacizumab plus temozolomide did not have better 12-month survival than patients assigned temozolomide monotherapy (59.8% vs 63.1%; p=not provided). This is a reversal of bevacizumab for patients with relapsed grade II and III glioma. | Roche Pharmaceuticals | Drug (targeted) | 9 |
| Coughlin et al  Lancet Oncology | Aug 2018 | Robot-assisted laparoscopic prostatectomy versus open radical retropubic prostatectomy: 24-month outcomes from a randomised controlled study | Robot-assisted laparoscopic prostatectomy was first introduced in 2001 and has become so widely practiced that recent estimates are that 90% of prostatectomies are done robotically.^84^ This method was thought to be superior to open surgery because of the less invasive nature, leading to less blood loss and easier recovery, albeit with higher financial costs and a need for more complex training. In a randomized trial where 326 men were randomized to laparoscopic prostatectomy or open radical retropubic prostatectomy, there were no differences in urinary function scores (88.68 [95% CI 86.79–90.58] vs 88.45 [86.54–90.36]; p<0.0001) or sexual function scores (International Index of Erectile Function Questionnaire: 29.75 [26.66–32.84] vs 29.78 [26.41–33.16], p<0.0001) at 6 months post-surgery between the two groups. This is a reversal of robot-assisted laparoscopic prostatectomy in men with prostate cancer. | Cancer Council Queensland | Procedure | 17 |
| Herbst et al  Lancet Oncology | Jan 2018 | Cetuximab plus carboplatin and paclitaxel with or without bevacizumab versus carboplatin and paclitaxel with or without bevacizumab in advanced NSCLC (SWOG S0819): a randomised, phase 3 study | It is well understood that non-small cell lung cancer (NSCLC) is a cancer with poor prognosis and few good treatment options. Results from a randomized trial showed a marginal but statistically significant improvement in survival with the addition of cetuximab to standard chemotherapy,^85^ and because of these results, cetuximab was added to the American Society of Clinical Oncology's list of potential treatment options for people with NSCLC.^85^ However, people have questioned whether the results support the use of cetuximab, especially in light of the $80,000-plus price tag for treatment.^86^ More recently, another randomized trial was performed to test the effectiveness of cetuximab in this setting. They found that among the 1333 patients assigned to cehmotherapy and either cetuximab or not, overall survival was no better among those assigned to cetuximab (HR 0.93, 95% CI 0.83–1.04; p=0.22), and PFS was no better in those with a positive in EGFR fluorescence in-situ hybridisation-positive cancers (HR 0.92, 95% CI 0.75–1.12; p=0.40). This is a reversal of the addition of cetuximab to chemotherapy in patients with NSCLC. | National Cancer Institute and Eli Lilly and Company | Drug (targeted | 8 |
| Vilgrain et al  Lancet Oncology | Dec 2017 | Efficacy and safety of selective internal radiotherapy with yttrium-90 resin microspheres compared with sorafenib in locally advanced and inoperable hepatocellular carcinoma (SARAH): an open-label randomised controlled phase 3 trial | Selective internal radiation therapy (SIRT) is a modality of delivering 90yttrium microspheres to liver tumors, while minimizing radiation hepatitis. Several microspheres are commercially available, and one, while not fully FDA approved, has received humanitarian device exemption for the treatment of hepatocellular carcinoma.^87^ In the SARAH Trial, patients assigned to SIRT (n=237; 8 months) had no better overall survival compared to those assigned to sorafenib (n=222; 9.9 months; HR=1·15 [95% CI=0·94–1·41]), thus suggesting that SIRT is not a better option for patients with hepatocellular carcinoma. This is a reversal of SIRT for patients with hepatocellular carcinoma. | Sirtex Medical Inc | Radiation | 15 |
| Tjan-Heijnen et al  Lancet Oncology | Nov 2017 | Extended adjuvant aromatase inhibition after sequential endocrine therapy (DATA): a randomised, phase 3 trial | Tamoxifen treatment for 5 years has long been used in the adjuvant setting of breast cancer. Aromatase inhibitors, when replacing tamoxifen after 2-3 years of treatment, were found to improve patient outcomes. The question then began to be about how long to treat patients with tamoxifen. Guidelines suggest that postmenopausal women with hormone receptor positive breast cancer be treated with either aromatase inhibitors or sequential tamoxifen followed by an aromatase inhibitor for a total duration of 5 years, or extended adjuvant endocrine therapy for a total of 10 years in patients initially treated with 5 years of tamoxifen.^88,89^ Prior to the guideline, some physicians had already been treating women with aromatase inhibitors for an extended period of time.^90^ In this trial, aromatase inhibition for 6 years (n=957) beyond tamoxifen treatment did not improve 5-year disease recurrence in postmenopausal women, compared to the standard 2-3 years tamoxifen followed by 3 years of aromatase inhibition (n=955; 83.1%; vs 79.4%; p=0.66). This is a reversal of aromatase inhibition for longer than 5 years for postmenopausal women with hormone receptor breast cancer. | AstraZeneca | Drug (hormone) | 2 |
| Seddon et al  Lancet Oncology | Oct 2017 | Gemcitabine and docetaxel versus doxorubicin as first-line treatment in previously untreated advanced unresectable or metastatic soft-tissue sarcomas (GeDDiS): a randomised controlled phase 3 trial | Doxorubicin in the first-line treatment for locally advanced or metastatic soft-tissue sarcoma has been standard of care for several decades. In the early 2000's, the combination of gemcitabine and docetaxel was proposed as an alternative to doxorubicin. Some physicians even began to use this combination as a first-line therapy for certain sarcomas.^91^ In the GeDDis trial, patients assigned to receive gemcitabine and docetaxel (n=128) did not have improved survival or disease-free survival than those who were assigned doxorubicin (n=129; HR=1.28; 95% CI=0.99 to 1.65; p=0.06). In fact, the intervention was almost harmful, suggesting that the combination of gemcitabine and docetaxel should not replace doxorubicin as standard of care for patients with sarcoma. This is a reversal of the combination of gemcitabine and docetaxel for patients with sarcoma. | Cancer Research UK, Sarcoma UK, and Clinical Trial Unit Kantonsspital St Gallen | Drug (cytotoxic) | 1 |
| Takahashi et al  Lancet Oncology | May 2017 | Prophylactic cranial irradiation versus observation in patients with extensive-disease small-cell lung cancer: a multicentre, randomised, open-label, phase 3 trial | Prophylactic cranial irradiation (PCI) was shown to improve outcomes in patients with small-cell lung cancer (SCLC). When the results of a large randomized control trial (European Organisation for Research and Treatment of Cancer (EORTC)) showed benefit in patients with extensive SCLC, the practice was adopted and incorporated into guidelines,^92^ but some questioned the methodology of the study. Another large study was conducted in Japan that assigned 113 patients to PCI and 111 to observation. They found that those that were assigned PCI did not have improved survival (11.6 months) compared to those assigned to observation (13.7 months; p=0.094), consequently showing a reversal of the PCI practice. This is a reversal of PCI for patients with SCLC. | The Ministry of Health, Labour and Welfare of Japan | Radiation | 10 |
| Dikmans et al  Lancet Oncology | Feb 2017 | Two-stage implant-based breast reconstruction compared with immediate one-stage implant-based breast reconstruction augmented with an acellular dermal matrix: an open-label, phase 4, multicentre, randomised, controlled trial | Most breast reconstruction surgeries after mastectomy are considered implant-based breast reconstruction (IBBR), which can be done either one-stage or two-stage. Historically, the two-stage method was most commonly used because it was thought that the subpectoral pocket after mastectomy was too small for the implant. With the addition of acelluar dermal matrices (ADMs), which minimize mastectomy flap contraction, the use of the one-stage method has increased dramatically, especially since it is thought to produce a more natural look.^93^ A randomized trial comparing these two methods (69 assigned to one-stage, 73 assigned to two-stage IBBR) found that surgical complications (46% vs. 18%; p=0.008) and reoperations for medical reasons (37% vs. 15%; p=0.14) were higher in the one-stage IBBR than in the two-stage IBBR. This is a reversal of the practice of one-stage IBBR for women undergoing implant-based beast reconstruction as part of breast cancer treatment. | Pink Ribbon, Nuts-Ohra, and LifeCell | Optimization | 5 |
| Clive et al  Lancet Oncology | Aug 2016 | Prophylactic radiotherapy for the prevention of procedure-tract metastases after surgical and large-bore pleural procedures in malignant pleural mesothelioma (SMART): a multicentre, open-label, phase 3, randomised controlled trial | Malignant mesothelioma, which has a poor prognosis, requires treatment that can disrupt the pleural cavity and cause procedure tract metastases (PTMs). It has been thought that prophylactic radiotherapy would help reduce these PTMs.^94^ This practice has been used for at least 20 years and by about 75% of oncology centers in the UK,^95^ and has been recommended by some organizations.^96^ In the SMART trial, patients assigned to immediate radiotherapy (n=102) did not have a lower incidence of PTMs than patients assigned to deferred radiotherapy (n=101; 9% vs. 16%; p=0.14). This is a reversal of prophylactic radiotherapy for patients with malignant pleural mesothelioma. | Research for Patient Benefit Programme from the UK National Institute for Health Research | Radiation | 8 |
| Glover et al  Lancet Oncology | Feb 2016 | Hyperbaric oxygen for patients with chronic bowel dysfunction after pelvic radiotherapy (HOT2): a randomised, double-blind, sham-controlled phase 3 trial | Hyperbaric oxygen was first used in the 1970s to treat patients with radiation tissue injury to the jawbone and has been used since to treat injury after pelvic radiation.^97^ The rationale is that because rapidly growing cells of the digestive tract are more sensitive to radiation, high oxygen concentrations delivered with hyperbaric oxygen could limit vessel scarring and improve tissue recovery.^97^ In 2005, this therapy was integrated into medical recommendations and approved by the FDA, despite randomized trials to support its efficacy.^98^ The HOT2 trial randomized patients to hyperbaric oxygen (n=55) or sham control (n=29). The investigators reported no difference in Inflammatory Bowel Disease Questionnaires between the hyperbaric oxygen and sham (4 vs. 4; p=0.50). This is a reversal of hyperbaric oxygen to prevent bowel dysfunction after pelvic radiotherapy. | Cancer Research UK and National Health Service (NHS) funding to the National Institute of Health Research Biomedical Research Centre at The Royal Marsden and the Institute of Cancer Research | Procedure | 5 |
| Stahel et al  Lancet Oncology | Dec 2015 | Neoadjuvant chemotherapy and extrapleural pneumonectomy of malignant pleural mesothelioma with or without hemithoracic radiotherapy (SAKK 17/04): a randomised, international, multicentre phase 2 trial | Extrapleural pneumonectomy is a common surgical procedure in the treatment and management of mesothelioma. Resected specimens often show mesothelioma in the resected margins, so postoperative radiotherapy has been practiced to provide treatment for this possibility, even though there is no evidence supporting its use.^99-101^ In a randomized trial, patients assigned to either hemithoracic radiotherapy or no radiotherapy (n=27 in each arm), median locoregional relapse-free survival from surgery was 7.6 months in the no radiotherapy group and 9.4 months in the radiotherapy group. This is a reversal of radiotherapy for patients with mesothelioma. | Swiss Group for Clinical Cancer Research, Swiss State Secretariat for Education, Research and Innovation, Eli Lilly | Radiation | 16 |
| Le Tourneau et al  Lancet Oncology | Oct 2015 | Molecularly targeted therapy based on tumour molecular profiling versus conventional therapy for advanced cancer (SHIVA): a multicentre, open-label, proof-of-concept, randomised, controlled phase 2 trial | In the era of precision medicine, drugs targeting specific molecular agents have been developed. Technology to identify genetic alterations in a timely manner has also been developed and is available to the public. The availability of these technologies has resulted in physicians to test tumors and treat cancer patients with drugs that target the tumor's genetic alterations, sometimes using off-label molecularly targeted drugs that have not been tested for efficacy for their respective indication.^102-104^ The SHIVA trial assigned patients with at least one molecular alteration to either a molecular-targeted regimen (n=99; experimental group) or treatment with physician's choice drug (n=96; control group). After follow-up there was no difference in median progression free survival between the experimental group and the control group (2.3 months vs. 2.0 months; p=0.41). This is a reversal of using molecularly targeted drugs outside of their indications. | Institut Curie | Optimization | 4 |
| Badwe et al  Lancet Oncology | Oct 2015 | Locoregional treatment versus no treatment of the primary tumour in metastatic breast cancer: an open-label randomised controlled trial | It is commonly thought that if a person has cancer, it should be removed. In the case of breast cancer, locoregional treatment is recommended for nonmetastatic breast cancer,^105^ but whether to perform locoregional treatment in metastatic breast cancer has come into question most recently. This practice was performed regularly, and retrospective and observational studies supported its use.^106,107^ However, evidence began to emerge that removal of the primary tumor in animal models could increase metastatic spread.^108^ In a randomized trial, median overall survival was no different between women with metastatic breast cancer assigned to locoregional treatment after chemotherapy (n=173) and women assigned to no locoregional treatment (n=177; 19.2 months vs. 20.5 months; p=0.79). This is a reversal of locoregional treatment in women with metastatic breast cancer who have had chemotherapy. | Department of Atomic Energy, Government of India | Procedure | 4 |
| Moss et al  Lancet Oncology | Sep 2015 | Effect of mammographic screening from age 40 years on breast cancer mortality in the UK Age trial at 17 years' follow-up: a randomised controlled trial | Breast cancer screening has been practiced for over 50 years and is recommended by several notable guideline organizations.^109^ Of course, the thought is that if a cancer is detected earlier, it can be treated earlier and both survival and prognosis are better for the patients. Recently, there has been concern that breast cancer screening is only detecting small tumors that would have not become pathogenic if not treated, thus resulting in an overdiagnosis of cancer. A randomized trial in the UK was implemented that assigned 53,883 women 39-41 years of age to the intervention arm (offered annual screening by mammography) and 106,953 women to the control arm (usual care). Breast cancer mammography screening only reduced breast cancer mortality in the first 10 years of the trial, but did not reduce mortality in the overall trial (absolute risk reduction per 1000 women was 0.32 (-0.38 to 1.02)). This is a reversal of population-based breast cancer screening with mammography in women 39-41 years. | National Institute for Health Research Health Technology Assessment programme and the American Cancer Society. Past funding was received from the Medical Research Council, Cancer Research UK, the UK Department of Health, and the US National Cancer Institute | Screening | 50 |
| Henderson et al., Lancet Oncology | Sep 2015 | Adjuvant lymph-node field radiotherapy versus observation only in patients with melanoma at high risk of further lymph-node field relapse after lymphadenectomy (ANZMTG 01.02/TROG 02.01): 6-year follow-up of a phase 3, randomised controlled trial | The lymph node field is a common site for relapse in melanoma, and adjuvant radiotherapy has been associated with fewer relapses. Because of this observation and with the motivation to not miss out on a potentially beneficial treatment, physicians have widely recommended adjuvant radiotherapy to the lymph node field.^110^ In a randomized trial where patients with high-risk melanoma and who had had a lymphadenectomy were assigned to adjuvant radiotherapy (n=123) or to no radiotherapy (n=127), overall survival (HR 1.27 [95% CI 0.89–1.79], p=0.21) and relapse-free survival (0.89 [0.65–1.22], p=0.51) were not different between groups. This is a reversal of routine adjuvant radiotherapy for patients with melanoma who are at high risk of relapse. | National Health and Medical Research Council of Australia, Cancer Council Australia, Melanoma Institute Australia, and the Cancer Council South Australia | Radiation | 17 |
| Soria et al  Lancet Oncology | Aug 2015 | Gefitinib plus chemotherapy versus placebo plus chemotherapy in EGFR-mutation-positive non-small-cell lung cancer after progression on first-line gefitinib (IMPRESS): a phase 3 randomised trial | EGFR tyrosine-kinase inhibitors (TKIs), such as gefitinib, are standard first line treatments for patients with non-small-cell lung cancer (NSCLC) and an EGFR mutation. Eventually, tumors in these patients often develop resistance to TKIs, but further treatment options are limited and there is no standard of care. In retrospectively evaluating medical records of patients with relapse, some patients continue on with gefitinib treatment, even though resistance has occurred.^111,112^ Using gefitinib for recurrent NSCLC has been approved in Japan since 2002.^111^ The IMPRESS trial randomized 133 patients with EGFR mutation positive NSCLC who continued to progress despite TKI treatment to gefitinib and 132 to placebo. They found that the addition of gefitinib to treatment did not prolong progression-free survival in these patients (5.4 months in each group; p=0.27). This is a reversal of continuing gefitinib in patients with NSCLC who have progressed because of TKI resistance. | AstraZeneca | Drug (targeted) | 6 |
| Primrose et al  Lancet Oncology | May 2014 | Systemic chemotherapy with or without cetuximab in patients with resectable colorectal liver metastasis: the New EPOC randomised controlled trial | Cetuximab was approved by the FDA and EMA in 2004 to treat advanced metastatic colorectal cancer.^113^ While not approved in the adjuvant setting, because of its use in the metastatic setting, physicians have extrapolated the benefits of cetuximab and have used it in the adjuvant setting.^114^ The New EPOC trial found that, after randomizing 117 patients to chemotherapy alone and 119 patients to chemotherapy plus cetuximab, not only did the cetuximab group not have better outcomes, progression free survival was actually higher in the group receiving the cetuximab plus chemotherapy (14.1 months vs. 20.5 months; p=0.03). This is a reversal of cetuximab in combination with chemotherapy prior to surgery for patients with resectable colorectal liver metastasis. | Cancer Research UK | Drug (targeted) | 1 |
| Judson et al  Lancet Oncology | Apr 2014 | Doxorubicin alone versus intensified doxorubicin plus ifosfamide for first-line treatment of advanced or metastatic soft-tissue sarcoma: a randomised controlled phase 3 trial | Soft-tissue sarcomas are relatively rare and do not have a good prognosis. Treatment options have not improved much in recent decades. Doxorubicin was first studied in 1972 as an option for sarcomas, and ifosfamide was used as a treatment option about a decade later.^115^ Trials during the 1990s began experimenting with the combinations of the two drugs.^115^ Clinically, these drugs have been used for over 30 years.^116^ In a randomized trial of patients assigned to doxorubicin (n=228) or doxorubicin and ifosfamide (n=227), median overall survival was no better in the combination group (14.3 months) than in the doxorubicin only group (12.8 months; p=0.08). This is a reversal of the combination of doxorubicin and ifosfamide in patients with advanced or metastatic soft-tissue sarcoma. | Cancer Research UK, EORTC Charitable Trust, UK NHS, Canadian Cancer Society Research Institute, Amgen | Drug (cytotoxic) | 30 |
| Bosset et al  Lancet Oncology | Feb 2014 | Fluorouracil-based adjuvant chemotherapy after preoperative chemoradiotherapy in rectal cancer: long-term results of the EORTC 22921 randomised study | Adjuvant chemotherapy is recommended for patients with stage III rectal cancer in the US, Canada, Australia, and China, in the hopes that adjuvant treatment will destroy micrometastases that might remain after surgery.^117^ A randomized trial of 1011 patients found that adjuvant fluorouracil-based chemotherapy did not improve 10 year disease free survival (47.0% vs. 43.7%; p=0.29) or overall survival (51.8% vs. 48.4%; p=0.32), compared to surveillance. This is a reversal of fluorouracil-based adjuvant chemotherapy after preoperative chemoradiotherapy in rectal cancer. | EORTC, US National Cancer Institute, Programme Hospitalier de Recherche Clinique, Ligue contre le Cancer Comité du Doubs | Drug (cytotoxic) | 1 |
| Bath-Hextall et al  Lancet Oncology | Jan 2014 | Surgical excision versus imiquimod 5% cream for nodular and superficial basal-cell carcinoma (SINS): a multicentre, non-inferiority, randomised controlled trial | Basal cell carcinoma is a common carcinoma of the skin and is usually treated by removal with Moh's surgery. Imiquimod is a cream that was approved in 1999 for the treatment of external genital warts and was later approved in 2004 for the treatment of basal cell carcinoma,^118^ although its use for this indication came earlier.^119^ In a randomized trial of 254 patients assigned to imiquimod cream and 247 patients assigned surgery, fewer patients treated with imiquimod cream were treated successfully (84% vs. 98%; p<0.0001). This is a reversal of imiquimod 5% cream for nodular and superficial basal-cell carcinoma. | [Cancer Research UK](https://www.sciencedirect.com/topics/medicine-and-dentistry/cancer-research) | Procedure | 10 |
| Veronesi et al  Lancet Oncology | Dec 2013 | Intraoperative radiotherapy versus external radiotherapy for early breast cancer (ELIOT): a randomised controlled equivalence trial | Radiotherapy is often used in conjunction with breast conserving surgery to minimize local recurrence. External radiotherapy has been the method most commonly performed but intraoperative radiotherapy is a newer but used technique.^120^ In the ELIOT trial, 654 patients were assigned to external radiotherapy and 651 patients were assigned to intraoperative radiotherapy. Patients assigned to intraoperative radiotherapy had higher ipsilateral breast tumor recurrence than external radiotherapy (4.4% vs 0.4%; p=0.0001). This is a reversal of intraoperative radiotherapy in patients with early breast cancer. | Italian Association for Cancer Research, Jacqueline Seroussi Memorial Foundation for Cancer Research, and Umberto Veronesi Foundation | Optimization | 18 |
| Garassino et al  Lancet Oncology | Sep 2013 | Erlotinib versus docetaxel as second-line treatment of patients with advanced non-small-cell lung cancer and wild-type EGFR tumours (TAILOR): a randomised controlled trial | At the time of the TAILOR trial, there were three standard treatment options for non-small cell lung cancer (NSCLC) - docetaxel, pemetrexed, and erlotinib. EGFR tyrosine kinase inhibitors, such as erlotinib, are the treatment of choice for in second and third lines of treatment, regardless of whether EGFR mutations are detected.^121,122^ Because the effect of EGFR tyrosine kinase inhibitors varies according to EGFR mutation status, the TAILOR trial randomized 110 patients to docetaxel and 112 patients to erlotinib. Patients assigned to erlotinib had shorter overall survival than those assigned to docetaxel (5.4 months vs. 8.2 months; p=0.05). This is a reversal of erlotinib as a second-line treatment of patients with NSCLLC and wild-type EGFR tumors. | Agenzia Italiana del Farmaco | Drug (targeted) | 9 |
| Kreissman et al  Lancet Oncology | Sep 2013 | Purged versus non-purged peripheral blood stem-cell transplantation for high-risk neuroblastoma (COG A3973): a randomised phase 3 trial | In situations where patients require an autologous hematopoietic stem cell transplantation, the practice of removing of selected cells (tumor purging) has become regularly practiced in the hopes of removing cells that could potentially cause relapse of certain types of cancers such as neuroblastoma.^123^ The practice began about 30 years ago,^124^ but there are several types of tumor purging done now.^123^ In a randomized trial with 243 patients assigned to receive purged peripheral blood stem cells and 229 patients assigned to non-purged peripheral blood stem cells, five-year event free survival was 40% in the purged group and 36% in the non-purged group (p=0.77). The results suggest a reversal of purging of peripheral blood stem cells for patients with high-risk neuroblastoma. | National Cancer Institute and Alex's Lemonade Stand Foundation | Procedure | 16 |
| van Hooft et al  Lancet Oncology | Apr 2011 | Colonic stenting versus emergency surgery for acute left-sided malignant colonic obstruction: a multicentre randomised trial | Self-expanding metal stents were developed in the 1990s for cases of inoperable gastrointestinal malignancy but have been used in cases of colorectal obstruction that requires surgery but is thought to reduce the need for emergency surgery.^125^ At the time of this study, there were three stents approved by the FDA.^126^ In a randomized trial of 47 patients assigned to colonic stenting and 51 patients assigned emergency surgery, mean global health status was no different in the colonic stenting group than in the emergency surgery group (63.0 vs. 61.4; p=0.36). Moreover, there was no difference in overall survival and a higher rate of bowel perforations in the stenting group, which could lead to seeding of malignant cells. This is a reversal of colonic stenting in patients with acute left-sided malignant colonic obstruction. | None | Procedure | 6 |
| Irani et al  Lancet Oncology | Feb 2010 | Efficacy of venlafaxine, medroxyprogesterone acetate, and cyproterone acetate for the treatment of vasomotor hot flushes in men taking gonadotropin-releasing hormone analogues for prostate cancer: a double-blind, randomised trial | Androgen deprivation therapy is commonly used in the treatment of men with prostate cancer, and often leads to symptomatic hot flashes. Hot flashes in men are often treated using therapies that have been well-studied in women but have little evidence on their effectiveness in men. These can either include hormonal (medroxyprogesterone acetate or cyproterone acetate) or non-hormonal treatments (venlafaxine).^127^ A trial randomized 919 men with prostate cancer and being treated with gonadotropin-releasing hormone to venlafaxine, medroxyprogesterone acetate, or cyproterone acetate. The change in median daily hot-flush score between randomization and 1 month  was –47.2% (IQR –74.3 to –2.5) in the venlafaxine group, –94.5% (–100.0 to –74.5) in the cyproterone group, and –83.7% (–98.9 to –64.3) in the medroxyprogesterone group, with the cyproterone and medroxyprogesterone groups with significantly better outcomes (p<0.001 for both groups compared to venlafaxine). This is a reversal of venlafaxine in preventing hot flashes in men with prostate cancer being treated with gonadotropin-releasing hormone. | Takeda Laboratories, Puteaux, France | Drug (other) | 8 |
| Le Péchoux et al  Lancet Oncology | May 2009 | Standard-dose versus higher-dose prophylactic cranial irradiation (PCI) in patients with limited-stage small-cell lung cancer in complete remission after chemotherapy and thoracic radiotherapy (PCI 99-01, EORTC 22003-08004, RTOG 0212, and IFCT 99-01): a randomised clinical trial | Based upon results from a meta-analysis showing the beneficial effects of prophylactic cranial irradiation (PCI) in prolonging survival in patients with small cell lung cancer (SCLC),^128,129^ this practice became standard. Standard radiation dose is 24 Gy, but based on some studies, 36 Gy is now recomended by some as the preferred dose.^128^ In a trial where 720 patients with SCLC and who were in complete remission after chemotherapy and thoracic radiotherapy were randomized to a 24 Gy standard dose or a higher 36 Gy radiation dose, there was no significant difference in the 2-year incidence of brain metastases between the standard PCI dose group and the higher-dose group (hazard ratio [HR] 0.80; 95% CI: 0.57–1.11; p=0.18).^130^ This is a reversal of high-dose radiotherapy in patients who are in remission from SCLC after chemotherapy. | Institut Gustave-Roussy, Association pour la Recherche sur le Cancer, Programme Hospitalier de Recherche Clinique. The European Organisation for Research and Treatment of Cancer (EORTC) contribution to this trial was supported by the US National Cancer Institute | Optimization | 11 |
| Gridelli et al  JAMA Oncology | 12/2018 | Safety and Efficacy of Bevacizumab Plus Standard-of-Care Treatment Beyond Disease Progression in Patients With Advanced Non–Small Cell Lung Cancer The AvaALL Randomized Clinical Trial | Bevacizumab is currently first-line treatment for patients with nonsquamous non-small cell lung cancer (NSCLC). While treatment with the drug is generally stopped after disease progression, some will continue to prescribe bevacizumab alongside standard of care (SOC) in hopes to improve outcomes.^131^ Bevacizumab after progression for NSCLC has shown promise in retrospective studies^132^ and in other cancer types such as metastatic colorectal cancer ^133^ The AvaALL randomized trial compared the effects of SOC plus bevacizumab beyond progression (n= 245) versus SOC alone (n=240) in NSCLC and found that the addition of bevacizumab did not improve overall survival. Median overall survival in the SOC+ bevacizumab group was 11.9 months (90% CI, 10.21-13.7) compared to 10.2 months (90% CI, 8.6-11.9) in the SOC group (HR, 0.84; 90% CI, 0.71-1.00; p=0.104). This is a reversal of continuing bevacizumab after disease progression in patients with advanced NSCLC. | F. Hoffmann-La Roche Ltd | Drug (targeted) | 9 |
| Scragg et al  JAMA Oncology | 11/2018 | Monthly High-Dose Vitamin D Supplementation and Cancer Risk A Post Hoc Analysis of the Vitamin D Assessment Randomized Clinical Trial | Vitamin D deficiency has been associated with higher risk of cancer incidence and mortality in observational studies,^134^ and many have recommended Vitamin D supplementation on the basis of these associations.^135,136^ To test this hypothesis, this trial randomized adults aged 50 to 84 years to receive high-dose Vitamin D (n=2558) or placebo (n=2552) and incidence of primary invasive and in situ malignant neoplasms were measured. The vitamin D group had a 6.5% incidence compared to 6.4% in the placebo group, a hazard ratio of 1.1 (95% CI, 0.81-1.25; p=.95). This is a reversal of high-dose vitamin D supplementation for preventing cancer in adults. | Health Research Council of New Zealand and by the Accident Compensation Corporation of New Zealand | Supplement/dietary | 13 |
| Yap et al  JAMA Oncology | 11/2017 | Predictors of Hand-Foot Syndrome and Pyridoxine for Prevention of Capecitabine–Induced Hand-Foot Syndrome A Randomized Clinical Trial | Capecitabine, a fluropyrimidine chemotherapy agent, frequently causes hand-foot syndrome (HFS) in patients.^137^ HFS causes swelling, pain, and redness on palms of hands and soles of feet, reducing quality of life and limiting daily activities. Pyridoxine, also known as B6 vitamin, is commonly prescribed to patients with HFS caused by chemotherapy.^138,139^ The pathophysiology of HFS is largely unknown, but anecdotal and retrospective studies found an association between pyridoxine and HFS relief.^140^ This study compared the effects of oral pyridozine (n=105) versus placebo (n=105) in patients receiving capecitabine on HFS incidence. The study found that there was no difference in outcomes between the groups, with HFS (grade two or higher) occurring in 33 patients (31.4%) in the pyridoxine group versus 39 patients (37.1%) in the placebo group (p=0.38). This is a reversal of pyridoxine for the prevention of capecitabine-induced HFS. | Singapore Cancer Society and the National Research Foundation Singapore | Supplement/dietary | 10 |
| Pramanik et al  JAMA Oncology | 9/2017 | Metronomic Chemotherapy vs Best Supportive Care in Progressive Pediatric Solid Malignant Tumors A Randomized Clinical Trial | Metronomic chemotherapy is a low-dose, long-lasting therapy with no prolonged breaks that is offered for patients with terminal disease.^141^ Metronomics can refer to both chemotherapeutic and nonchemotherapeutic agents to slow tumor growth and reduce toxic effects compared regular doses and regimens. Metronomic chemotherapy has been in practice since 2000, but its use is mostly based on retrospective and small, unrandomized trials.^142,143^ This study investigated the effects of metronomic chemotherapy consisting of thalidomide, celecoxib, etoposide, and cyclophosphamide in pediatric patients with primary extracranial, nonhematopoeitic solid malignant tumors that progressed after at least 2 lines of chemotherapy and had no further curative options. The 4-drug oral metronomic regimen (n=56) did not significantly prolong PFS or OS compared to placebo (n=52). At 2.9 months, 96.4% of patients had disease progression in the metronomic group versus 100% in the placebo group (p=0.24). Median PFS between groups was similar (HR, 0.69; 95% CI, 0.47-1.03; P=.07), as was median OS (HR, 0.74; 95% CI, 0.50-1.09; p=0.13). This is a reversal of metronomic chemotherapy in pediatric patients with extracranial progressive solid malignant tumors with no further curative options. | PallCanCare, a nongovernmental organization dedicated to palliative care. | Procedure | 17 |

1. Cooper JS, Pajak TF, Forastiere AA, et al. Postoperative Concurrent Radiotherapy and Chemotherapy for High-Risk Squamous-Cell Carcinoma of the Head and Neck. 2004;350(19):1937-1944.

2. Tanvetyanon T, Padhya T, McCaffrey J, et al. Postoperative concurrent chemotherapy and radiotherapy for high‐risk cutaneous squamous cell carcinoma of the head and neck. 2015;37(6):840-845.

3. Mok TS, Kim S-W, Wu Y-L, et al. Gefitinib plus chemotherapy versus chemotherapy in epidermal growth factor receptor mutation-positive non-small-cell lung cancer resistant to first-line gefitinib (IMPRESS): overall survival and biomarker analyses. 2017;35(36):4027-4034.

4. Inomata M, Shukuya T, Takahashi T, et al. Continuous administration of EGFR-TKIs following radiotherapy after disease progression in bone lesions for non-small cell lung cancer. 2011;31(12):4519-4523.

5. Maruyama R, Wataya H, Seto T, Ichinose YJAr. Treatment after the failure of gefitinib in patients with advanced or recurrent non-small cell lung cancer. 2009;29(10):4217-4221.

6. Chambers SK, Occhipinti S, Foley E, et al. Mindfulness-based cognitive therapy in advanced prostate cancer: a randomized controlled trial. 2016;35(3):291-297.

7. Abdollahi F, Khan MS. Role of Mindfulness‐Based Cognitive Therapy in Alleviating Psychological Distress among Cancer Patients. 2014.

8. Bartley T. *Mindfulness-based cognitive therapy for cancer: Gently turning towards.* John Wiley & Sons; 2011.

9. Demeestere I, Brice P, Peccatori FA, et al. No evidence for the benefit of gonadotropin-releasing hormone agonist in preserving ovarian function and fertility in lymphoma survivors treated with chemotherapy: final long-term report of a prospective randomized trial. 2016;34(22):2568-2574.

10. Slater C, Liang M, McCune J, Christman G, Laufer MJL. Preserving ovarian function in patients receiving cyclophosphamide. 1999;8(1):3-10.

11. Shin SWJKjor. The current practice of transarterial chemoembolization for the treatment of hepatocellular carcinoma. 2009;10(5):425-434.

12. Dhanasekaran R, Kooby DA, Staley CA, Kauh JS, Khanna V, Kim HSJJoso. Comparison of conventional transarterial chemoembolization (TACE) and chemoembolization with doxorubicin drug eluting beads (DEB) for unresectable hepatocelluar carcinoma (HCC). 2010;101(6):476-480.

13. Van Hazel GA, Heinemann V, Sharma NK, Peeters MJJoco-NY. SIRFLOX: randomized phase III trial comparing first-line mFOLFOX6 (plus or minus bevacizumab) versus mFOLFOX6 (plus or minus bevacizumab) plus selective internal radiation therapy in patients with metastatic colorectal cancer. 2016;34(15):1723-1731.

14. Welsh JS, Kennedy AS, Thomadsen BJIJoROBP. Selective internal radiation therapy (SIRT) for liver metastases secondary to colorectal adenocarcinoma. 2006;66(2):S62-S73.

15. Van Hazel G, Blackwell A, Anderson J, et al. Randomised phase 2 trial of SIR‐Spheres® plus fluorouracil/leucovorin chemotherapy versus fluorouracil/leucovorin chemotherapy alone in advanced colorectal cancer. *Journal of surgical oncology.* 2004;88(2):78-85.

16. Adams JR, Elting LS, Lyman GH, et al. Use of erythropoietin in cancer patients: assessment of oncologists' practice patterns in the United States and other countries. 2004;116(1):28-34.

17. Schneider G, Voltz R, Gaertner JJBC. Cancer pain management and bone metastases: an update for the clinician. 2012;7(2):113-120.

18. Prevention CfDCa. Cancer Survivorship Care Plans. *Cancer Survivors* 2018; https://[www.cdc.gov/cancer/survivors/life-after-cancer/survivorship-care-plans.htm](http://www.cdc.gov/cancer/survivors/life-after-cancer/survivorship-care-plans.htm). Accessed July 16, 2019.

19. Society AC. Survivorship Care Plans. *Survivorship: During and After Treatment* 2019; https://[www.cancer.org/treatment/survivorship-during-and-after-treatment/survivorship-care-plans.html](http://www.cancer.org/treatment/survivorship-during-and-after-treatment/survivorship-care-plans.html). Accessed July 16, 2019.

20. Pivot X, Manikhas A, Żurawski B, et al. CEREBEL (EGF111438): a phase III, randomized, open-label study of lapatinib plus capecitabine versus trastuzumab plus capecitabine in patients with human epidermal growth factor receptor 2–positive metastatic breast cancer. 2015;33(14):1564-1573.

21. Ryan Q, Ibrahim A, Cohen MH, et al. FDA drug approval summary: lapatinib in combination with capecitabine for previously treated metastatic breast cancer that overexpresses HER-2. 2008;13(10):1114-1119.

22. Pivot X, Manikhas A, Żurawski B, et al. CEREBEL (EGF111438): A Phase III, Randomized, Open-Label Study of Lapatinib Plus Capecitabine Versus Trastuzumab Plus Capecitabine in Patients With Human Epidermal Growth Factor Receptor 2–Positive Metastatic Breast Cancer. 2015;33(14):1564-1573.

23. Bachelot T, Romieu G, Campone M, et al. Lapatinib plus capecitabine in patients with previously untreated brain metastases from HER2-positive metastatic breast cancer (LANDSCAPE): a single-group phase 2 study. *The lancet oncology.* 2013;14(1):64-71.

24. Nersesyan H, Slavin KV. Current aproach to cancer pain management: Availability and implications of different treatment options. *Ther Clin Risk Manag.* 2007;3(3):381-400.

25. Swarm RA, Abernethy AP, Anghelescu DL, et al. Adult cancer pain. 2013;11(8):992-1022.

26. d'Amato TA, Galloway M, Szydlowski G, Chen A, Landreneau RJ. Intraoperative brachytherapy following thoracoscopic wedge resection of stage I lung cancer. *Chest.* Oct 1998;114(4):1112-1115.

27. Blasberg JD, Pass HI, Donington JS. Sublobar Resection: A Movement from the Lung Cancer Study Group. *Journal of Thoracic Oncology.* 2010/10/01/ 2010;5(10):1583-1593.

28. Melosky B, Agulnik J, Assi H. Retrospective practice review of treatment of metastatic non-small-cell lung cancer with second-line erlotinib. *Curr Oncol.* 2008;15(6):279-285.

29. Miller K, Steger GG, Niepel D, Lüftner DJPc, diseases p. Harnessing the potential of therapeutic agents to safeguard bone health in prostate cancer. 2018;21(4):461.

30. Smith MR, Halabi S, Ryan CJ, et al. Randomized Controlled Trial of Early Zoledronic Acid in Men With Castration-Sensitive Prostate Cancer and Bone Metastases: Results of CALGB 90202 (Alliance). 2014;32(11):1143-1150.

31. Saad F, McKiernan J, Eastham J. Rationale for zoledronic acid therapy in men with hormone-sensitive prostate cancer with or without bone metastasis. Paper presented at: Urologic Oncology: Seminars and Original Investigations2006.

32. Fried M. Octreotide in the treatment of refractory diarrhea. *Digestion.* 1999;60 Suppl 2:42-46.

33. Maroun JA, Anthony LB, Blais N, et al. Prevention and management of chemotherapy-induced diarrhea in patients with colorectal cancer: a consensus statement by the Canadian Working Group on Chemotherapy-Induced Diarrhea. *Curr Oncol.* 2007;14(1):13-20.

34. Hoff PM, Saragiotto DF, Barrios CH, et al. Randomized Phase III Trial Exploring the Use of Long-Acting Release Octreotide in the Prevention of Chemotherapy-Induced Diarrhea in Patients With Colorectal Cancer: The LARCID Trial. 2014;32(10):1006-1011.

35. Loprinzi CL, Qin R, Dakhil SR, et al. Phase III Randomized, Placebo-Controlled, Double-Blind Study of Intravenous Calcium and Magnesium to Prevent Oxaliplatin-Induced Sensory Neurotoxicity (N08CB/Alliance). 2014;32(10):997-1005.

36. Grothey A, Nikcevich DA, Sloan JA, et al. Intravenous calcium and magnesium for oxaliplatin-induced sensory neurotoxicity in adjuvant colon cancer: NCCTG N04C7. *J Clin Oncol.* 2011;29(4):421-427.

37. Gamelin L, Boisdron-Celle M, Delva R, et al. Prevention of oxaliplatin-related neurotoxicity by calcium and magnesium infusions: a retrospective study of 161 patients receiving oxaliplatin combined with 5-Fluorouracil and leucovorin for advanced colorectal cancer. 2004;10(12):4055-4061.

38. Field KM, Kosmider S, Jefford M, Jennens R, Green M, Gibbs P. Chemotherapy Treatments for Metastatic Colorectal Cancer: Is Evidence-Based Medicine in Practice? 2008;4(6):271-276.

39. Onsrud M, Kolstad P, Normann T. Postoperative external pelvic irradiation in carcinoma of the corpus stage I: A controlled clinical trial. *Gynecologic Oncology.* 1976/06/01/ 1976;4(2):222-231.

40. Keller D, Kempson RL, Levine G, McLennan CJC. Management of the patient with early endometrial carcinoma. 1974;33(4):1108-1116.

41. Mandell L, Nori D, Anderson L, Hilaris B. Postoperative vaginal radiation in endometrial cancer using a remote afterloading technique. *International Journal of Radiation Oncology*Biology*Physics.* 1985/03/01/ 1985;11(3):473-478.

42. Hollingworth W, Metcalfe C, Mancero S, et al. Are Needs Assessments Cost Effective in Reducing Distress Among Patients With Cancer? A Randomized Controlled Trial Using the Distress Thermometer and Problem List. 2013;31(29):3631-3638.

43. NCCN Clinical Practice Guidelines in Oncology: Distress Management (ed 3) 2012 National Comprehensive Cancer Network Fort Washington PNCCN. *NCCN Clinical Practice Guidelines in Oncology: Distress Management.* Fort Washington, PA: National Comprehensive Cancer Network

2012.

44. Rosenberg SA. The management of Hodgkin's disease: Half a century of change: The Kaplan Memorial Lecture. *Annals of Oncology.* 1996;7(6):555-560.

45. Hematology ASo. 50 years in hematology: Reserach that revolutionized patient care. 2008.

46. Li B, Yu J, Suntharalingam M, et al. Comparison of three treatment options for single brain metastasis from lung cancer. 2000;90(1):37-45.

47. Khan AJ, Dicker AP. On the Merits and Limitations of Whole-Brain Radiation Therapy. 2013;31(1):11-13.

48. Bruera E, Hui D, Dalal S, et al. Parenteral Hydration in Patients With Advanced Cancer: A Multicenter, Double-Blind, Placebo-Controlled Randomized Trial. 2013;31(1):111-118.

49. Dunlop R, Ellershaw J, Baines M, Sykes N, Saunders CJJome. On withholding nutrition and hydration in the terminally ill: has palliative medicine gone too far? A reply. 1995;21(3):141-143.

50. Cruciani RA, Zhang JJ, Manola J, Cella D, Ansari B, Fisch MJ. L-Carnitine Supplementation for the Management of Fatigue in Patients With Cancer: An Eastern Cooperative Oncology Group Phase III, Randomized, Double-Blind, Placebo-Controlled Trial. 2012;30(31):3864-3869.

51. ACOB TEITELBAUM M. Acetyl L-Carnitine (ALC) for CFS, Cancer Related Fatigue and Fibromyalgia. *Vitality101 with Dr.T*2012.

52. Grunfeld E, Julian JA, Pond G, et al. Evaluating Survivorship Care Plans: Results of a Randomized, Clinical Trial of Patients With Breast Cancer. 2011;29(36):4755-4762.

53. Ganz PA, Hewitt M. *Implementing cancer survivorship care planning: workshop summary.* National Academies Press; 2007.

54. Ganz PA, Casillas J, Hahn EE. Ensuring Quality Care for Cancer Survivors: Implementing the Survivorship Care Plan. *Seminars in Oncology Nursing.* 2008/08/01/ 2008;24(3):208-217.

55. Knight K, Wade S, Balducci LJTAjom. Prevalence and outcomes of anemia in cancer: a systematic review of the literature. 2004;116(7):11-26.

56. Seidenfeld J, Piper M, Flamm C, et al. Epoetin Treatment of Anemia Associated With Cancer Therapy: a Systematic Review and Meta-analysis of Controlled Clinical Trials. *JNCI: Journal of the National Cancer Institute.* 2001;93(16):1204-1214.

57. Dunst J. The use of epoetin alfa to increase and maintain hemoglobin levels during radiotherapy. Paper presented at: Seminars in oncology2001.

58. Rose PG. Endometrial Carcinoma. 1996;335(9):640-649.

59. Scholten AN, van Putten WL, Beerman H, et al. Postoperative radiotherapy for Stage 1 endometrial carcinoma: long-term outcome of the randomized PORTEC trial with central pathology review. 2005;63(3):834-838.

60. Sneed PK, Lamborn KR, Forstner JM, et al. Radiosurgery for brain metastases: is whole brain radiotherapy necessary? 1999;43(3):549-558.

61. Regine WF, Huhn JL, Patchell RA, et al. Risk of symptomatic brain tumor recurrence and neurologic deficit after radiosurgery alone in patients with newly diagonised brain metastases: results and implications. 2002;52(2):333-338.

62. Bohlius J, Wilson J, Seidenfeld J, et al. Erythropoietin or Darbepoetin for patients with cancer. *Cochrane Database of Systematic Reviews.* 2006(3).

63. Aapro MS, Link HJTO. September 2007 update on EORTC guidelines and anemia management with erythropoiesis-stimulating agents. 2008;13(Supplement 3):33-36.

64. Rizzo JD, Somerfield MR, Hagerty KL, et al. Use of epoetin and darbepoetin in patients with cancer: 2007 American Society of Clinical Oncology/American Society of Hematology clinical practice guideline update. 2008;26(1):132-149.

65. Clark AS, Vahdat LTJSct. Chemotherapy-induced palmar-plantar erythrodysesthesia syndrome: etiology and emerging therapies. 2004;1(4):213-218.

66. Ademola J, Frazier C, Kim SJ, Theaux C, Saudez XJAjocd. Clinical evaluation of 40% urea and 12% ammonium lactate in the treatment of xerosis. 2002;3(3):217-222.

67. Pendharkar D, Goyal H. Novel & effective management of capecitabine induced Hand Foot Syndrome. 2004;22(14_suppl):8105-8105.

68. Lacouture ME, Wu S, Robert C, et al. Evolving strategies for the management of hand–foot skin reaction associated with the multitargeted kinase inhibitors sorafenib and sunitinib. 2008;13(9):1001-1011.

69. Abushullaih S, Saad ED, Munsell M, Hoff PMJCi. Incidence and severity of hand–foot syndrome in colorectal cancer patients treated with capecitabine: a single-institution experience. 2002;20(1):3-10.

70. Mortimer JE, Lauman MK, Tan B, Dempsey CL, Shillington AC, Hutchins KSJJoOPP. Pyridoxine treatment and prevention of hand-and-foot syndrome in patients receiving capecitabine. 2003;9(4):161-166.

71. Kirkwood JM, Strawderman MH, Ernstoff MS, Smith TJ, Borden EC, Blum RHJJoco. Interferon alfa-2b adjuvant therapy of high-risk resected cutaneous melanoma: the Eastern Cooperative Oncology Group Trial EST 1684. 1996;14(1):7-17.

72. Sosman J. Adjuvant therapy for cutaneous melanoma. 2019; https://[www.uptodate.com/contents/adjuvant-therapy-for-cutaneous-melanoma#H157827560](http://www.uptodate.com/contents/adjuvant-therapy-for-cutaneous-melanoma#H157827560). Accessed July 16, 2019.

73. Kirkwood JM, Tarhini AA, Moschos SJ, Panelli MCJNRCO. Adjuvant therapy with high-dose interferon α2b in patients with high-risk stage IIB/III melanoma. 2007;5(1):2.

74. Gridelli C, Maione P, Castaldo V, Rossi A. Gefitinib in elderly and unfit patients affected by advanced non-small-cell lung cancer. *Br J Cancer.* 2003;89(10):1827-1829.

75. Stahel R, Rossi A, Petruzelka L, et al. Lessons from the ("Iressa" Expanded Access Programme: gefitinib in special non-small-cell lung cancer patient populations. *Br J Cancer.* 2003;89 Suppl 2(Suppl 2):S19-S23.

76. Griffin JP, Koch KA, Nelson JE, Cooley MEJC. Palliative care consultation, quality-of-life measurements, and bereavement for end-of-life care in patients with lung cancer: ACCP evidence-based clinical practice guidelines. 2007;132(3):404S-422S.

77. Kuerer HMJTLO. More evidence for further minimisation of breast-cancer surgery. 2018;19(10):1272-1273.

78. Giuliano AE, Kirgan DM, Guenther JM, Morton DL. Lymphatic mapping and sentinel lymphadenectomy for breast cancer. *Annals of surgery.* Sep 1994;220(3):391-398; discussion 398-401.

79. Chao AHJPSN. A review of the use of acellular dermal matrices in postmastectomy immediate breast reconstruction. 2015;35(3):131-134.

80. Ibrahim AMS, Koolen PGL, Ashraf AA, et al. Acellular Dermal Matrix in Reconstructive Breast Surgery: Survey of Current Practice among Plastic Surgeons. *Plast Reconstr Surg Glob Open.* 2015;3(4):e381-e381.

81. van den Bent MJ, Klein M, Smits M, et al. Bevacizumab and temozolomide in patients with first recurrence of WHO grade II and III glioma, without 1p/19q co-deletion (TAVAREC): a randomised controlled phase 2 EORTC trial. 2018;19(9):1170-1179.

82. Nghiemphu PL, Liu W, Lee Y, et al. Bevacizumab and chemotherapy for recurrent glioblastoma: a single-institution experience. *Neurology.* 2009;72(14):1217-1222.

83. Chamberlain MC, Johnston S. Bevacizumab for recurrent alkylator-refractory anaplastic oligodendroglioma. 2009;115(8):1734-1743.

84. Tsui C, Klein R, Garabrant MJSe. Minimally invasive surgery: national trends in adoption and future directions for hospital strategy. 2013;27(7):2253-2257.

85. Pirker R, Pereira JR, Szczesna A, et al. Cetuximab plus chemotherapy in patients with advanced non-small-cell lung cancer (FLEX): an open-label randomised phase III trial. *Lancet (London, England).* May 2 2009;373(9674):1525-1531.

86. Fojo T, Grady C. How much is life worth: cetuximab, non-small cell lung cancer, and the $440 billion question. *J Natl Cancer Inst.* 2009;101(15):1044-1048.

87. Stubbs RS, Wickremesekera SK. Selective internal radiation therapy (SIRT): a new modality for treating patients with colorectal liver metastases. *HPB.* 2004/09/01/ 2004;6(3):133-139.

88. Burstein HJ, Temin S, Anderson H, et al. Adjuvant endocrine therapy for women with hormone receptor-positive breast cancer: american society of clinical oncology clinical practice guideline focused update. *J Clin Oncol.* Jul 20 2014;32(21):2255-2269.

89. Senkus E, Kyriakides S, Ohno S, et al. Primary breast cancer: ESMO Clinical Practice Guidelines for diagnosis, treatment and follow-up. *Annals of oncology : official journal of the European Society for Medical Oncology.* Sep 2015;26 Suppl 5:v8-30.

90. Whelan TJ, Pritchard KIJCcr. Managing patients on endocrine therapy: focus on quality-of-life issues. 2006;12(3):1056s-1060s.

91. Seddon B, Strauss SJ, Whelan J, et al. Gemcitabine and docetaxel versus doxorubicin as first-line treatment in previously untreated advanced unresectable or metastatic soft-tissue sarcomas (GeDDiS): a randomised controlled phase 3 trial. *The Lancet Oncology.* 2017/10/01/ 2017;18(10):1397-1410.

92. Slotman BJJTLO. Time to reconsider prophylactic cranial irradiation in extensive-stage small-cell lung cancer? 2017;5(18):566-567.

93. Macadam SA, Lennox PA. Acellular dermal matrices: Use in reconstructive and aesthetic breast surgery. *Can J Plast Surg.* Summer 2012;20(2):75-89.

94. Stewart SA, Clive AO, Maskell NA, Penz EJPo. Evaluating quality of life and cost implications of prophylactic radiotherapy in mesothelioma: Health economic analysis of the SMART trial. 2018;13(2):e0190257.

95. Lee C, Bayman N, Swindell R, Faivre-Finn CJLc. Prophylactic radiotherapy to intervention sites in mesothelioma: a systematic review and survey of UK practice. 2009;66(2):150-156.

96. Thorax BTSSoCCJ. BTS statement on malignant mesothelioma in the UK, 2007. 2007;62(Suppl 2):ii1.

97. medicine VMcfh. Radiation Tissue Injury.

98. Services AH. HYPERBARIC OXYGEN THERAPY FOR LATE RADIATION TISSUE INJURY IN CERVICAL AND OTHER GYNECOLOGICAL MALIGNANCIES *CLINICAL PRACTICE GUIDELINE GYNE-003* 2009. https://[www.albertahealthservices.ca/assets/info/hp/cancer/if-hp-cancer-guide-gyne003-hbot-for-lrti.pdf](http://www.albertahealthservices.ca/assets/info/hp/cancer/if-hp-cancer-guide-gyne003-hbot-for-lrti.pdf).

99. Rintoul R, Treasure T, Macbeth F. Multimodal treatment for malignant pleural mesothelioma. 2015.

100. Parker C, Neville EJT. Lung cancer• 8: Management of malignant mesothelioma. 2003;58(9):809-813.

101. Butchart EGJTO. Contemporary management of malignant pleural mesothelioma. 1999;4(6):488-500.

102. Krzyzanowska MK. Off-label use of cancer drugs: a benchmark is established. *J Clin Oncol.* Mar 20 2013;31(9):1125-1127.

103. Gorski D. Dr. Stanislaw Burzynski’s “personalized gene-targeted cancer therapy”: Can he do what he claims for cancer? *Science-Based Medicine.* December 5, 2011, 2011.

104. De Souza JA, Polite B, Perkins M, et al. Unsupported off-label chemotherapy in metastatic colon cancer. 2012;12(1):481.

105. Mauriac L, Luporsi E, Cutuli B, et al. Summary version of the Standards, Options and Recommendations for nonmetastatic breast cancer (updated January 2001). *Br J Cancer.* 2003;89 Suppl 1(Suppl 1):S17-S31.

106. Tubiana M, Koscielny S. The natural history of breast cancer and the link between local recurrence and distant metastases: implications for therapy. *Reports of Practical Oncology & Radiotherapy.* 2001/01/01/ 2001;6(4):181-195.

107. Juan O, Lluch A, de Paz L, et al. Prognostic factors in patients with isolated recurrences of breast cancer (stage IV-NED). *Breast cancer research and treatment.* Jan 1999;53(2):105-112.

108. Demicheli R, Retsky MW, Swartzendruber DE, Bonadonna G. Proposal for a new model of breast cancer metastatic development. *Annals of oncology : official journal of the European Society for Medical Oncology.* Nov 1997;8(11):1075-1080.

109. Fletcher SW. Breast Cancer Screening: A 35-Year Perspective. *Epidemiologic Reviews.* 2011;33(1):165-175.

110. Brady MS. Adjuvant radiation for patients with melanoma. *The Lancet Oncology.* 2015/09/01/ 2015;16(9):1003-1004.

111. Watanabe S, Tanaka J, Ota T, et al. Clinical responses to EGFR-tyrosine kinase inhibitor retreatment in non-small cell lung cancer patients who benefited from prior effective gefitinib therapy: a retrospective analysis. January 01 2011;11(1):1.

112. Maruyama R, Wataya H, Seto T, Ichinose Y. Treatment after the failure of gefitinib in patients with advanced or recurrent non-small cell lung cancer. *Anticancer research.* Oct 2009;29(10):4217-4221.

113. Merck KGaA: Erbitux Approved for Launch in European Union. *Business Wire.* June 30, 2004, 2004.

114. Fong YJTLO. Adjuvant therapy after hepatectomy for colorectal metastases. 2014;15(6):544-545.

115. Spira AI, Ettinger DSJTo. The use of chemotherapy in soft-tissue sarcomas. 2002;7(4):348-359.

116. Judson I, Verweij J, Gelderblom H, et al. Doxorubicin alone versus intensified doxorubicin plus ifosfamide for first-line treatment of advanced or metastatic soft-tissue sarcoma: a randomised controlled phase 3 trial. 2014;15(4):415-423.

117. Beets GL, Glimelius BLJTlo. Adjuvant chemotherapy for rectal cancer still controversial. 2014;15(2):130-131.

118. Bubna AK. Imiquimod - Its role in the treatment of cutaneous malignancies. *Indian J Pharmacol.* Jul-Aug 2015;47(4):354-359.

119. Smith KJ, Germain M, Yeager J, Skelton H. Topical 5% imiquimod for the therapy of actinic cheilitis. *Journal of the American Academy of Dermatology.* 2002/10/01/ 2002;47(4):497-501.

120. Harrison LB, Enker WEJO. High-dose-rate intraoperative radiation therapy for colorectal cancer. 1995;9(7).

121. Garassino MC, Martelli O, Broggini M, et al. Erlotinib versus docetaxel as second-line treatment of patients with advanced non-small-cell lung cancer and wild-type EGFR tumours (TAILOR): a randomised controlled trial. 2013;14(10):981-988.

122. Jassem J, Dziadziuszko RJTLO. EGFR inhibitors for wild-type EGFR NSCLC: to use or not to use? 2013;14(10):916-917.

123. Lowdell MW, Theocharous PJTo. “Less is More”: the role of purging in hematopoietic stem cell transplantation. 1997;2(4):268-274.

124. Klingebiel TJTLO. Role of purging in PBSC transplantation for neuroblastoma. 2013;14(10):919.

125. Kim EJ, Kim YJ. Stents for colorectal obstruction: Past, present, and future. *World J Gastroenterol.* 2016;22(2):842-852.

126. Baron TH. Colonic Stenting: Technique, Technology, and Outcomes for Malignant and Benign Disease. *Gastrointestinal Endoscopy Clinics of North America.* 2005/10/01/ 2005;15(4):757-771.

127. Irani J, Salomon L, Oba R, Bouchard P, Mottet NJTlo. Efficacy of venlafaxine, medroxyprogesterone acetate, and cyproterone acetate for the treatment of vasomotor hot flushes in men taking gonadotropin-releasing hormone analogues for prostate cancer: a double-blind, randomised trial. 2010;11(2):147-154.

128. Gregor A. Prophylactic cranial irradiation in small-cell lung cancer: is it ever indicated? *Lung Cancer.* 1998;12(1).

129. Lally BE, Urbanic JJ, Blackstock AW. Could it be that less is more? *The Lancet Oncology.* 2009;10(5):435-437.

130. Le Péchoux C, Dunant A, Senan S, et al. Standard-dose versus higher-dose prophylactic cranial irradiation (PCI) in patients with limited-stage small-cell lung cancer in complete remission after chemotherapy and thoracic radiotherapy (PCI 99-01, EORTC 22003-08004, RTOG 0212, and IFCT 99-01): a randomised clinical trial. *The lancet oncology.* 2009;10(5):467-474.

131. Leon L, Kosty M, Jahanzeb M, et al. Effectiveness of bevacizumab exposure beyond disease progression in patients with non–small‐cell lung cancer: analyses of the ARIES observational cohort study. 2016;25(5):569-577.

132. Kosty MP, Wozniak AJ, Jahanzeb M, et al. Effectiveness and safety of post-induction phase bevacizumab treatment for patients with non-small-cell lung cancer: results from the ARIES observational cohort study. *Targeted oncology.* Dec 2015;10(4):509-516.

133. Grothey A, Flick ED, Cohn AL, et al. Bevacizumab exposure beyond first disease progression in patients with metastatic colorectal cancer: analyses of the ARIES observational cohort study. *Pharmacoepidemiology and drug safety.* Jul 2014;23(7):726-734.

134. Afzal S, Brondum-Jacobsen P, Bojesen SE, Nordestgaard BG. Genetically low vitamin D concentrations and increased mortality: Mendelian randomisation analysis in three large cohorts. *Bmj.* Nov 18 2014;349:g6330.

135. Gorham ED, Garland CF, Garland FC, et al. Vitamin D and prevention of colorectal cancer. *The Journal of steroid biochemistry and molecular biology.* 2005;97(1-2):179-194.

136. Vieth R. Why the optimal requirement for Vitamin D3 is probably much higher than what is officially recommended for adults. *The Journal of Steroid Biochemistry and Molecular Biology.* 2004/05/01/ 2004;89-90:575-579.

137. Twelves C, Wong A, Nowacki MP, et al. Capecitabine as Adjuvant Treatment for Stage III Colon Cancer. 2005;352(26):2696-2704.

138. Gressett SM, Stanford BL, Hardwicke F. Management of hand-foot syndrome induced by capecitabine. 2006;12(3):131-141.

139. Yap Y-S, Kwok L-L, Syn N, et al. Predictors of Hand-Foot Syndrome and Pyridoxine for Prevention of Capecitabine–Induced Hand-Foot Syndrome: A Randomized Clinical TrialPyridoxine for Capecitabine–Induced Hand-Foot SyndromePyridoxine for Capecitabine–Induced Hand-Foot Syndrome. *JAMA Oncology.* 2017;3(11):1538-1545.

140. Vukelja SJ, Lombardo FA, James WD, Weiss RB. Pyridoxine for the palmar-plantar erythrodysesthesia syndrome. *Annals of internal medicine.* Oct 15 1989;111(8):688-689.

141. Porkholm M, Toiviainen-Salo S, Seuri R, et al. Metronomic therapy can increase quality of life during paediatric palliative cancer care, but careful patient selection is essential. 2016;105(8):946-951.

142. Pramanik R, Agarwala S, Gupta YK, et al. Metronomic Chemotherapy vs Best Supportive Care in Progressive Pediatric Solid Malignant Tumors: A Randomized Clinical TrialMetronomic Chemotherapy vs Supportive Care in Solid Malignant TumorsMetronomic Chemotherapy vs Supportive Care in Solid Malignant Tumors. *JAMA Oncology.* 2017;3(9):1222-1227.

143. André N, Carré M, Pasquier E. Metronomics: towards personalized chemotherapy? *Nature Reviews Clinical Oncology.* 06/10/online 2014;11:413.
